# Genomic prediction in early selection stages using multi-year data in a hybrid rye breeding program

**DOI:** 10.1186/s12863-017-0512-8

**Published:** 2017-05-31

**Authors:** Angela-Maria Bernal-Vasquez, Andres Gordillo, Malthe Schmidt, Hans-Peter Piepho

**Affiliations:** 1 Biostatistics Unit, Institute of Crop Science, University of Hohenheim, Fruwirthstrasse 23, Stuttgart, 70599 Germany; 2 KWS-LOCHOW GMBH, Ferdinand-von-Lochow-Strasse 5, Bergen, 29303 Germany

**Keywords:** Multi-year data, Genomic prediction, Genotype-by-year interaction, Hybrid rye breeding

## Abstract

**Background:**

The use of multiple genetic backgrounds across years is appealing for genomic prediction (GP) because past years’ data provide valuable information on marker effects. Nonetheless, single-year GP models are less complex and computationally less demanding than multi-year GP models. In devising a suitable analysis strategy for multi-year data, we may exploit the fact that even if there is no replication of genotypes across years, there is plenty of replication at the level of marker loci. Our principal aim was to evaluate different GP approaches to simultaneously model genotype-by-year (*GY*) effects and breeding values using multi-year data in terms of predictive ability. The models were evaluated under different scenarios reflecting common practice in plant breeding programs, such as different degrees of relatedness between training and validation sets, and using a selected fraction of genotypes in the training set. We used empirical grain yield data of a rye hybrid breeding program. A detailed description of the prediction approaches highlighting the use of kinship for modeling *GY* is presented.

**Results:**

Using the kinship to model *GY* was advantageous in particular for datasets disconnected across years. On average, predictive abilities were 5% higher for models using kinship to model *GY* over models without kinship. We confirmed that using data from multiple selection stages provides valuable *GY* information and helps increasing predictive ability. This increase is on average 30% higher when the predicted genotypes are closely related with the genotypes in the training set. A selection of top-yielding genotypes together with the use of kinship to model *GY* improves the predictive ability in datasets composed of single years of several selection cycles.

**Conclusions:**

Our results clearly demonstrate that the use of multi-year data and appropriate modeling is beneficial for GP because it allows dissecting *GY* effects from genomic estimated breeding values. The model choice, as well as ensuring that the predicted candidates are sufficiently related to the genotypes in the training set, are crucial.

**Electronic supplementary material:**

The online version of this article (doi:10.1186/s12863-017-0512-8) contains supplementary material, which is available to authorized users.

## Background

Genomic prediction (GP) is a tool for predicting genomic estimated breeding values (GEBV) of selection candidates based on marker information. A reference set of individuals, called training set (TS), is phenotyped and genotyped to train a model, which can be used to predict GEBV of another set of individuals that has only been genotyped but not phenotyped, the so-called prediction or validation set (VS) [1]. Prediction performance of GP procedures can be assessed through cross validation (GP-CV). In GP-CV the datasets are divided into *k* folds, where *k*-1 folds are used for model training and the remaining fold for model validation. This process is repeated using each of the *k* folds in turn as validation set and then repeating the process several times. An alternative method to evaluate prediction performance is genomic prediction - forward validation (GP-FV), which makes use of data from previous years for training the model to predict genotypes tested in later years and in this way validate the model. GP-FV mimics the ultimate goal in plant breeding, where new genotypes in new environments are to be predicted.

One of the factors determining the accuracy of the predictions is the size of the training and the validation set [2–5]; thus, using multi-year data is an attractive approach to train GP procedures because it allows increasing the TS-size, thereby potentially increasing prediction performance. But using multi-year data is challenging because different cycles (in different years) are disconnected, that is, there are no genotypes in common across cycles; therefore, genotype-by-year effects (*GY*) and genotype main effects will be confounded. The only connection across years is genetic, i.e., through the relatedness within the material, which we expect, since the data comes from a breeding program. The genetic connectivity has been difficult to exploit with standard phenotypic models. Multi-location field trial data in breeding programs are often analyzed by year and not over years because: (i) it is simpler and faster, and (ii) it is difficult to accurately estimate variation across years, partly because few if any genotypes are common between breeding cycles. If *GY* effects are not properly modeled, the genomic prediction procedure will divert part of the marker information into prediction of the *GY* interaction effects rather than the GEBV. This situation poses the main challenge when combining data across years.

Several authors have proposed an extension of the GP model to predict genotype-by-environment interaction effects by incorporating environmental data and crop modeling [6, 7] or assuming a covariance matrix composed of a genotype-related and an environment-related component [8, 9]. In these studies, environment is understood as the conditions of a given location in a given year, i.e., the conditions in a year-location combination, and no attempt is made to differentiate the effects of locations and years. Hence, year-location combinations are represented by a single factor for “environment”. In the structure of the present hybrid rye breeding program, however, it is crucial to separate the location and year effects, since the program runs in the same locations across years and the interest of the breeders is in predicting the GEBV free of *GY* and genotype-by-location (*GL*) effects. Most procedures used for GP do not include model terms that dissect genotype effects, including GEBV and *GY*, mainly because of the lack of overlapping genotypes across years (selection cycles in the TS).

We hypothesize that in a multi-year dataset of a breeding program, where there are no common genotypes across years, GEBV can be dissected from *GY* based on the genetic correlation between genotypes via the kinship matrix. Further, genotypes from the same breeding cycle evaluated in multiple years in the TS will enhance the separation between GEBV and *GY* effects. In light of this, our principal objective was to evaluate the merit of different models accounting for the *GY* effect. In order to put the different models to a realistic test, we evaluated them under scenarios representing common practice in breeding programs, i.e., in different relatedness scenarios and top-yield selection scenarios, where different fractions of genotypes with top-yield performance in the TS were selected. The top-yield selection scenarios are interesting to breeders because considering only subsets of the best genotypes would allow reducing the effect of genotypes with confounded yield- and non-yield-QTL effects, i.e., genotypes whose grain yield is susceptible to be affected by diseases or lodging or other - environmentally triggered - threshold traits.

## Methods

### Phenotypic data structure

**Fig. 1:**
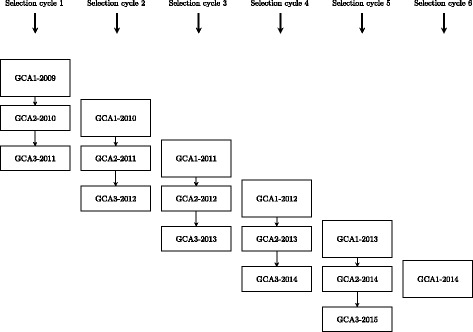
Selection cycles structure in the rye hybrid breeding program

A first stage of the present hybrid rye program consists of selfing single plants and selecting for line *per se* performance in the subsequent selfing generations. After line *per se* evaluation, selected lines are crossed to one or more single crosses from the opposite gene pool. The testcross progenies are evaluated in multi-location trials [10] to assess their general combining ability (GCA). In the first year of testcross evaluations, *S*_2_ lines are evaluated, from which a selected fraction is subjected to a more intensive evaluation in the following year (GCA2), across a larger number of environments. Again, a selected fraction of genotypes is carried forward to a third selection stage (GCA3), where genotypes are evaluated in more environments and with more testers (See Additional file 1: Figure S1 for a complete selection cycle description). The minimum generation interval comprises five years, which is the time from initial crossing to GCA1. In Fig. 1, we depict the breeding program structure to define the different GP-FV scenarios.

New GCA1 experiments are carried out each year with new testers from the opposite gene pool, whereas testers remain the same across GCA1 and GCA2 experiments within the same selection cycle. At KWS-LOCHOW, a selected fraction of genotypes are test-crossed for GCA3 in combination with a different set of testers compared to GCA1 and GCA2, whereas the candidates are a selected fraction of the candidates in GCA1 and GCA2. GCA1 experiments of different selection cycles (e.g. GCA1-2009, GCA1-2010, GCA1-2011) do not normally share any genotype or check entry. Further, a GCA experiment consists of multi-environment trials (METs), where subsets of genotypes are evaluated in series of trials allocated in several locations (in one year). Within a year, trials are connected through common genotypes and check entries. Trials are laid out as *α*-designs with two replicates and 32 incomplete blocks of size 12 to 16.

We analyzed grain yield data from two rye hybrid breeding programs located in Germany and Poland of KWS-LOCHOW. Three datasets were formed, i.e., the German (GER) dataset, with only German lines, the Polish (PL) dataset, with only Polish lines, and the pooled dataset with German and Polish lines (GER&PL). The datasets were screened for outliers at the trial level using the method BH-MADR developed in Bernal-Vasquez et al. [11]. The genotype sets evaluated at the GCA1 level differ between the two breeding programs within the same year. When selected candidates reach the GCA2 and GCA3 stage, they are evaluated in one common trial series across locations. We used a GP-FV approach, where GEBV of a VS with genotypes not included in the TS are predicted. We considered three scenarios that differ in the composition of their TS, different relatedness scenarios between TS and VS, and additionally, two different selection fractions for the set of top-yielding genotypes. To assess prediction performance we computed the predictive abilities of each scenario in the three datasets, i.e., GER, PL and GER&PL. Predictive abilities are defined in Subsection *Predictive abilities* of this Section.

In the scenarios described in the following, the use of GCA1, GCA2 and GCA3 data may indirectly increase the proportion of segregating first-degree relatives in the TS in comparison to a control TS composed of only GCA1 data. Each scenario is composed of three VS, one complete TS and a control TS (Additional file 1: Figures S2–S4). The VS were: VS_1_: GCA1-2012, VS_2_: GCA1-2013 and VS_3_: GCA1-2014. The control TS scenarios do not include the GCA2 and GCA3 trials. In the control TS, GCA1 data do not share common genotypes at all, thus we can evaluate if using kinship to model *GY* indeed helps to dissect *GY* from GEBV, thus allowing a more accurate predictive ability. Complete TS make use of all data in the cycle in order to check whether having this additional information about some genotypes across the years also allows to better dissect *GY* from GEBV with or without the use of kinship to model the *GY* effects. This comparison between control TS and complete TS is important because by using control TS we loose information of the common genotypes evaluated in additional years. In the complete TS, we exploit the information of those overlapping genotypes, which are very few in the end (approx. 1 to 2% in GCA3 from the total in GCA1), but we can evaluate by cross validation whether they are sufficient to improve the estimate of the *GY* effect. Since the minimum generation interval in the breeding scheme from crossing to GCA1 is five years, one would need to have breeding cycles going back at least five years to include parental lines in the TS. Hence, it is assumed that, for example, genotypes selected in GCA1-2009 are most likely to be the parents of genotypes evaluated in GCA1-2014. Thus, GCA1-2014 is likely to be more closely related to GCA1-2009 than GCA1-2013 to GCA1-2009. This theoretical relatedness cannot always be realized, as the parental lines can be renewed any time or kept longer in the program. With this in mind, many TS-VS combinations can be evaluated as interesting scenarios, some being more realistic than others. Keeping the TS fixed to evaluate different VS in different years is more convenient for comparing predictive abilities, acknowledging that some TS-VS scenarios may not seem entirely realistic in that prediction is backwards rather than forwards in time. We would hold, however, that temporal direction is not crucial when evaluating predictive accuracy of a model or method.

The first scenario comprises lines from one selection cycle and corresponds to data from GCA1-2009, GCA2-2010, GCA3-2011 as TS (TS_1_) to predict VS_1_, VS_2_ and VS_3_ (Additional file 1: Figure S2). The control set corresponds to GCA1-2009 (controlTS_1_).

The second scenario comprises lines of two selection cycles with data from GCA1-2009, GCA2-2010 (from selection cycle 1), GCA1-2010 and GCA2-2011 (from selection cycle 2) as TS (TS_2_) to predict VS_1_, VS_2_ and VS_3_ (Additional file 1: Figure S3). As control TS we use GCA1-2009 and GCA1-2010 (controlTS_2_).

The third scenario comprises lines of three selection cycles with data from GCA1-2009, GCA2-2010, GCA3-2011 (of selection cycle 1), GCA1-2010, GCA2-2011, GCA3-2012 (of selection cycle 2), and GCA1-2011, GCA2-2012, GCA3-2013 (of selection cycle 3) as TS (TS_3_) to predict VS_1_, VS_2_, and VS_3_ (Additional file 1: Figure S4). The control TS contains GCA1-2009, GCA1-2010 and GCA1-2011 (controlTS_3_).

To verify our hypothesis that using the kinship matrix helps to separate the GEBV from *GY* effects, we evaluated four different models using the complete TS (explained in the following) plus two models using the control TS of each scenario. The models were evaluated in three relatedness situations for each of the above described scenarios: all available genotypes (All-scenario) and genotypes with no (0P-scenario) and with one (1P-scenario) parent in the TS. The TS-size remains fixed and the VS-size changes according the relatedness degree with the TS. To guarantee a fair comparison with VS of the same size for the All-, 0P- and 1P-scenarios, a simple random sampling was carried out to ensure VS-size of 100 genotypes. We ran 10 iterations for VS-size =100 and computed the simple means and confidence intervals of the estimated predictive abilities. The scenarios for the GER dataset with VS_1_ used VS-size = 90, since there were less than 100 available genotypes. Finally, different selection fractions of top-yielding genotypes in the TS were evaluated TS composed of the 100% (Top100%), 75% (Top75%) and 50% (Top50%) best yielding genotypes, i.e., TS-sizes vary and VS-sizes remain fixed including all available genotypes with markers.

### Genotypic data

The marker information was obtained using a 10K Infinium iSelect HD Custom BeadChip (Illumina, San Diego, CA, USA). Monomorphic markers and markers with minor allele frequency (MAF) less than 1% or missing information of more than 10% per marker were dropped. A total of 10633 markers passed the quality test and were used for GP. Homozygous marker genotypes were coded as -1 and 1, and the heterozygous type, missing values and technical failures were coded as 0 [12–14].

### Statistical models for the training sets

Mixed models are widely used for multi-environment trial (MET) analysis and can be fitted either in a single stage or in multiple stages. A single-stage analysis models the entire observed data in one stage at the level of individual plots, whereas a stage-wise analysis splits the analysis into analyses at the level of factors that are hierarchically nested, e.g., first by environments and then across environments [15].

The single-stage model can be stated as

1 $$  \gamma = T : G \times Y \times L + T \cdot (G \times Y \times L) + (Y \cdot L)/S/R/B + e,  $$

where *γ* is the vector of observed genotype yields, *G* represents the genotypes, *T* the testers, *Y* the years, *L* the locations, *S* the trials within locations, *R* the replicates within trials, *B* the blocks within replicates, and *e* the error associated with the observation *γ*. In the statement of model (1), we have used the notation described in Piepho et al. [16], where the dot operator (·) defines crossed effects (*A*·*B*), the crossing operator (×) defines a full factorial model (*A*×*B*=*A*+*B*+*A*·*B*) and the nesting operator (/) indicates that a factor *B* is nested within another factor *A* (*A*/*B*=*A*+*A*·*B*). The colon (:) is used to separate fixed (first) from random effects (last). Our model (1) takes all factors except *T* as random. It is therefore resolved as

2 $$\begin{array}{*{20}l}  \gamma &= T : G + Y + L + G \cdot Y + G \cdot L + Y \cdot L + G \cdot Y \cdot L \\ &\quad+ G \cdot T + T \cdot Y + T \cdot L + G \cdot T \cdot Y + G \cdot T \cdot L \\ &\quad+ T \cdot Y \cdot L + G \cdot T \cdot Y \cdot L + Y \cdot L \cdot S + Y \cdot L \cdot S \cdot R\\ &\quad+ Y \cdot L \cdot S \cdot R \cdot B + e. \end{array} $$

In routine analysis of breeding trials, it is common to analyze the data in stages. For this reason, we here also consider different stage-wise approaches. The following models are stage-wise representations of the single-stage model (1). They differ in the number of stages and the assumptions to model *GY*. As will become apparent, there are several options for stage-wise analysis and it is not obvious which option is preferable regarding our main objective to dissect *GY* from GEBV effects, which is why we compare different approaches. In some models, we move *G* to the fixed part to enable estimation of genotype means, for example in the second stage, where we then submit the means to a third stage. It is stressed here that taking *G* as fixed during all stages except the last is just a technical requirement to render the stage-wise analysis equivalent to the single-stage analysis, and this does not change the status of the genotype factor as random in the full stage-wise analysis [15]. In the models where *G* is kept as fixed, we will have *T* and *G* in the fixed part of the model. The interaction *G*·*T* is taken as random because not all genotypes are testcrossed with the same testers and because, as just mentioned, *G* keeps its random status in the last stage.

Note the slightly different interpretations of the main effect *G* depending on the context. This effect refers in general to the genotypic main effect. In the GP stage, however, where it is modeled with the marker information (i.e. using kinship), the main effect *G* refers specifically to the pure additive genetic part of the genotypic effect, i.e. the GEBV.

Among the models used for the control and the complete datasets, some use kinship to model *GY* and others not. For clarity, we differentiate approaches used for the control TS (described first with labels A1 and A1K) from the approaches using complete TS (with labels A2, A3, A4 and A5). The distinction is to point out the difference in the connectivity between the control TS and the complete TS. The control TS do not share common genotypes across years, whereas the complete TS share a fraction of selected genotypes within selection cycles, i.e., across GCA1 + GCA2 + GCA3 of the same cycle. Approaches A2 and A3 are a two-stage version of model (1), whereas approaches A4 and A5 have three stages. In A2, A3 and A5 we use kinship to model *GY*, while for the A4 approach, kinship is not used to model *GY*. Table 1 summarizes the labels, the short notation (both used indistinctly to better link the approaches in the Discussion and the Figures) and a brief description with the key elements to distinguish the approaches. A detailed explanation of the models A1 to A5 follows next.

**Table 1:** Summary of GP-FV approaches

Label	Short notation	TS used	No. stages	Use of Kinship	Description
				to model *GY*	
A1	Year-wise	controlTS_1_, controlTS_2_,	2 + GP	no	Year-wise model and GP with year as fixed effect
	without kinship	controlTS_3_			
A1K	Year-wise	controlTS_2_, controlTS_3_	2 + GP	yes	Year-wise model and GP with year as fixed effect
	with kinship				and *GY* modeled using kinship
A2	2-stg-Kin	TS_1_, TS_2_, TS_3_	2	yes	Across years model with GP included in the 2nd
					stage and *GY* modeled using kinship
A3	2-stg-Kin-het	TS_1_, TS_2_, TS_3_	2	yes	Across years model with GP included in the 2nd
					stage and *GY* modeled using kinship. Allows heterogeneous
					rogeneous variance among years in the *GY* interaction effect
A4	3-stg-NoKin	TS_1_, TS_2_, TS_3_	3	no	Across years model for the TS using no kinship to model *GY*.
					Third stage is GP
A5	3-stg-Kin	TS_1_, TS_2_, TS_3_	3	yes	Across years model for the TS. Uses kinship in the 2nd stage
					of the TS to model *GY*. Third stage is GP

#### Year-wise approach without (A1) and with (A1K) kinship: modeling for the control sets

All the control TS are composed of independent GCA1 trials in one, two or three years (controlTS_1_, controlTS_2_ and controlTS_3_, respectively). We denote them as independent because the GCA1 trials have no checks in common. Thus, one approach was to estimate adjusted genotype means for each year separately in a first step and then model a fixed year effect while obtaining GEBV for genotypes in the GP stage [17]. This approach presumes that the mean of the genotypes evaluated in one year is a better year effect estimate than the year effect estimate based on a few checks shared across years. The approach is based on the assumption that the genotypes evaluated in each year are a random sample of the breeding population. Hereafter, we refer to this method as the year-wise approach (A1). One disadvantage of this approach is that it disregards annual genetic gain (1 to 2%).

In the first stage, we model the plot data within locations and years as

3 $$ \gamma = G \cdot T : S/R/B + e,   $$

which is resolved as

4 $$\begin{array}{*{20}l} \gamma = G \cdot T : S + S \cdot R + S \cdot R \cdot B + e,  \end{array} $$

where factors are defined as for model (1). Adjusted genotype-by-tester means (**m** ^(1)^) are computed for each year-location combination and are submitted to the second stage, where adjusted genotype means (**m** ^(2)^) are calculated, using a year-wise model defined as

5 $$\begin{array}{*{20}l}  \mathbf{m}^{(1)} = & G + T : G \cdot T + L \cdot (G \times T) + \epsilon_{1} \\  = & G + T : G \cdot T + L \cdot G + L \cdot T + L \cdot G \cdot T + \epsilon_{1}. \end{array} $$

All terms are defined as for model (1), *ε*_1_ is the vector of errors associated with the adjusted means **m** ^**(1)**^ with **ε**_**1**_∼*N*(**0**,**R**_**1**_) and **R**_**1**_ is a diagonal matrix whose diagonal elements are computed from the inverse of the variance-covariance matrix estimated in the first stage [18]. Hereafter, **m** ^**(x)**^ always denotes the adjusted mean and **R**_**x**_ always denotes a diagonal matrix carrying over these diagonal weights computed in the x −*t* *h* stage. The model at the GP stage is then

6 $$ \mathbf{m}^{\mathbf{(2)}} = \mathbf{X}\mathbf{\beta} + \mathbf{Z}_{\mathbf{g}} \mathbf{u}_{\mathbf{g}} + \epsilon_{\mathbf{2}},   $$

where **m** ^**(2)**^ is the vector of adjusted genotype means across years, **X** is the design matrix of the years, **β** is the vector of year effects, **Z**_**g**_ is the marker matrix for genotypes, and **u**_**g**_ the vector of marker effects. We assume that $\mathbf {u}_{\mathbf {g}} \sim N\left (\mathbf {0},\mathbf {I}\sigma ^{2}_{\mathrm {u}_{\mathrm {g}}}\right)$, and $var\left (\mathbf {Z}_{\mathbf {g}} \mathbf {u}_{\mathbf {g}}\right) = \mathbf {Z}_{\mathbf {g}} \mathbf {Z}_{\mathbf {g}}^{\mathbf {T}}\sigma ^{2}_{\mathrm {u}_{\mathrm {g}}}$. Furthermore, **ε**_**2**_ is the vector of errors associated with the adjusted means **m** ^**(2)**^ with *ε*_2_∼*N*(**0**,*R*_2_).

The alternative approach is to additionally model the *GY* effects in the GP stage. Hereafter, we refer to this strategy as the year-wise with kinship approach (A1K). Given the disconnectedness of the genotypes across years in GCA1 trials, dissecting the genotype main effects *G* (the GEBV) and the *GY* becomes difficult. If kinship information is included to model the genotypic correlation among relatives, it may be possible to dissect the *G* and *GY* effects, provided that genotypes tested in different years can be regarded as representative of the same breeding population, which is usually the case. A slight bias will be incurred though due to genetic progress, but this can be tolerated if more than outweighed by the improved precision of the year effect estimate. The key idea behind the use of kinship to dissect the *GY* effects is that, while there is no replication of genotypes across years, there is plenty of replication across years at the level of genes and their alleles.

The model for the GP is

7 $$ \mathbf{m}^{\mathbf{(2)}} = \mathbf{X}\beta + \mathbf{Z}_{\mathbf{g}} \mathbf{u}_{\mathbf{g}} + \mathbf{Z}_{\mathbf{gy}} \mathbf{u}_{\mathbf{gy}} + \epsilon_{\mathbf{2}},   $$

where **m** ^**(2)**^, **X** *β* and **Z**_**g**_ **u**_**g**_ are defined as for model (6). The *GY* effects are modeled as **w**=**Z**_**gy**_ **u**_**gy**_, with **Z**_**gy**_ as the marker matrix for genotypes-by-year effects and **u**_**gy**_ the vector of marker-by-year effects whose variance is $var\left (\mathbf {u}_{\mathbf {gy}}\right) = \mathbf {I}\sigma ^{2}_{\mathrm {u}_{\text {gy}}}$, and hence $var(\mathbf {w}) = \mathbf {Z}_{\mathbf {gy}} \mathbf {Z}_{\mathbf {gy}}^{T} \sigma ^{2}_{\mathrm {u}_{\text {gy}}}$.

In particular, **Z**_**gy**_ is a block-diagonal matrix with blocks given by the marker coefficient matrices of genotypes in a given year $\left (\mathbf {Z}_{\mathbf {gy}_{\mathbf {r}}}\right)$, e.g., $\mathbf {Z}_{\mathbf {gy}} =\left (\begin {array}{ccc} \mathbf {Z}_{\mathbf {gy}_{\mathbf {1}}} & \mathbf {0} & \mathbf {0} \\ \mathbf {0} & \mathbf {Z}_{\mathbf {gy}_{\mathbf {2}}} & \mathbf {0} \\ \mathbf {0} & \mathbf {0} & \mathbf {Z}_{\mathbf {gy}_{\mathbf {3}}} \end {array}\right)$.

Under the mixed model formulation of ridge regression, $\mathbf {Z}_{\mathbf {gy}} \mathbf {Z}_{\mathbf {gy}}^{\mathbf {T}} \sigma ^{2}_{\text {gy}}$ represents the linear structure of the genotype-by-year variance-covariance matrix with covariance of two genotypes within the same year depending on the similarity in their marker profiles [12]. Note that the covariance among different years is zero. Any covariance between years is captured by the main effect for genotypes via the **Z**_**g**_ matrix.

#### Two-stage approach with kinship matrix: 2-stg-Kin (A2)

The single-stage model (1) can be estimated in a two-stage analysis, where adjusted genotype-tester means by locations and years are computed in the first stage, and then in the second stage, adjusted genotype means across locations and years are calculated. GP-FV can be incorporated in this second stage, allowing to compute GEBVs for a set of genotypes that belong to a new year, i.e. the VS.

The first stage remains as for the previous approaches and is described by model (3). The second-stage model is

8 $$  \mathbf{m}^{\mathbf{(1)}} = T : G \times Y \times L + T \cdot (G \times Y \times L) + \epsilon_{1}.  $$

The model is fitted using the adjusted genotype-by-tester means **m** ^**(1)**^ for the different year-location combinations computed in the first stage. The four-way factorial in model (8) is resolved as

9 $$\begin{array}{*{20}l}  T &: G + Y + L + G \cdot Y + G \cdot L + Y \cdot L + T \cdot Y + T \cdot L \\ &+ G \cdot T + G \cdot Y \cdot L + G \cdot T \cdot Y + G \cdot T \cdot L + T \cdot Y\\ &\times L + G \cdot T \cdot Y \cdot L. \end{array} $$

Hence, the second-stage model (8) can be written as

10 $$  \mathbf{m}^{\mathbf{(1)}} = \mathbf{1}\mu + \mathbf{X}\beta + \mathbf{Z}_{\mathbf{g}} \mathbf{u}_{\mathbf{g}} + \mathbf{Z}_{\mathbf{gy}} \mathbf{u}_{\mathbf{gy}} + \mathbf{Z}_{\mathbf{b}} \mathbf{u}_{\mathbf{b}} + \epsilon_{\mathbf{1}},  $$

where **m** ^**(1)**^ is the vector of adjusted genotype-tester means obtained in the first stage [model (3)], **1** is a *m*×1 vector of ones with *m* the number of genotypes, **μ** is the intercept, **X** is the design matrix for fixed effects, **β** is the vector of fixed-effects parameters. The tester (*T*) is the only fixed effect in model (9). The GEBV *G* is equivalent to **v**=**Z**_**g**_ **u**_**g**_, with **Z**_**g**_ the marker matrix for genotypes and **u**_**g**_ the vector of marker effects whose variance is $var\left (\mathbf {u}_{\mathbf {g}}\right) = \mathbf {I} \sigma ^{2}_{\mathrm {u}_{\mathrm {g}}}$, and hence $var(\mathbf {v}) = \mathbf {Z}_{\mathbf {g}} \mathbf {Z}_{\mathbf {g}}^{\mathbf {T}} \sigma ^{2}_{\mathrm {u}_{\mathrm {g}}}$. Similarly, the genotype-by-year effect *G*·*Y* is equivalent to **w**=**Z**_**gy**_ **u**_**gy**_, where **Z**_**gy**_ is the marker matrix for genotypes-by-year and **u**_**gy**_ is the vector of marker-by-year effects whose variance is assumed to be $var\left (\mathbf {u}_{\mathbf {gy}}\right) = \mathbf {I}\sigma ^{2}_{\mathrm {u}_{\text {gy}}}$, then $var(\mathbf {w}) = \mathbf {Z}_{\mathbf {gy}}\mathbf {Z}_{\mathbf {gy}}^{\mathbf {T}} \sigma ^{{2}}_{{u}_{{gy}}}$. **Z**_**b**_ is the design matrix for the other random effects between years and **u**_**b**_ is the vector of random effects between years, which includes the effects of *G*×*Y*×*L*+*T*·(*G*×*Y*×*L*) except *G* and *G*·*Y*. Thus, $\mathbf {u}_{\mathbf {b}} = \left (\mathbf {u}^{\mathbf {T}}_{\mathbf {b}(\mathbf {1})}, \mathbf {u}^{\mathbf {T}}_{\mathbf {b}(\mathbf {2})}, \ldots, \mathbf {u}^{\mathbf {T}}_{\mathbf {b}(\mathbf {t})}\right)^{\text {T}}$ with **u**_**b**(**k**)_ the vector of the *k*-th random effect between years, and $var\left (\mathbf {u}_{\mathbf {b}}\right) = \mathbf {\Sigma }_{\mathbf {b}} = \oplus ^{\mathbf {t}}_{\mathbf {k} = \mathbf {1}} \mathbf {\Sigma }_{\mathbf {b}(\mathbf {k})}$ with $var\left (\mathbf {u}_{\mathbf {b}(\mathbf {k})}\right) = \mathbf {\Sigma }_{\mathbf {b}(\mathbf {k})} = \mathbf {I} \sigma ^{2}_{\mathrm {b(k)}}$. The symbol ⊕ denotes the direct sum of matrices and defines block diagonal matrices [19]. The vector of errors is **ε**_**1**_ with **ε**_**1**_∼*N*(**0**,**R**_**1**_).

#### Two-stage approach with kinship matrix and heterogeneous variance: 2-stg-Kin-het (A3)

In this approach, we allow heterogeneity among years in the variance of the interaction *G*·*Y*. Thus, for model (10) we assume $var\left (\mathbf {u}_{\mathbf {gy}}\right) = \mathbf {\Lambda } = \mathbf {\oplus }^{\mathbf {m}}_{\mathbf {r} = \mathbf {1}} \mathbf {I} \sigma ^{2}_{\text {u}_{\text {gy}_{\text {(r)}}}}$, where $\sigma ^{2}_{\text {u}_{\text {gy}_{\text {(r)}}}}$ is the genotype-by-year variance in the *r*-th year with the genotype entries sorted by year. If **w**=**Z**_**gy**_ **u**_**gy**_, then $var(\mathbf {w}) = \mathbf {Z}_{\mathbf {gy}} \Lambda \mathbf {Z}_{\mathbf {gy}}^{\mathbf {T}}$.

#### Three-stage approach without kinship: 3-stg-NoKin (A4)

A three-stage approach for GP-FV may alleviate the computational burden imposed by using a two-stage model. In practice, plant breeders often use the following three-stage approach: In the first stage adjusted genotype-tester means (**m** ^**(1)**^) are estimated per year-location combination using model (3). In the second stage adjusted genotype means across years and locations (**m** ^**(2)**^) are estimated using the model

11 $$ \mathbf{m}^{\mathbf{(1)}} = \mathbf{X} \mathbf{\beta} + \mathbf{Z}_{\mathbf{b}} \mathbf{u}_{\mathbf{b}} + \mathbf{\epsilon}_{\mathbf{1}},   $$

where **X** is the design matrix for fixed effects **β**. We need *G* to be fitted as a fixed effect (together with *T*), since we are estimating adjusted genotype means. Except for overlapping genotypes across different selection stages (GCA1, GCA2, GCA3), within the same selection cycles, the *G*·*Y* variance component is completely confounded with that for *G* under this model. **Z**_**b**_ and **u**_**b**_ are the design matrix and vector for the random effects between years, respectively. The vector includes all random effects indicated in model (8) except *G*. **u**_**b**_ is equivalent to $\left (\mathbf {u}^{\mathbf {T}}_{\mathbf {b(1)}}, \mathbf {u}^{\mathbf {T}}_{\mathbf {b(2)}}, \ldots, \mathbf {u}^{\mathbf {T}}_{\mathbf {b(t)}}\right)^{\text {T}}$ with **u**_**b(k)**_ the vector of the *k*-th random between-year effects. The variance is $var(\mathbf {u}_{\mathbf {b}}) = \mathbf {\Sigma }_{\mathbf {b}} = \mathbf {\oplus }^{\mathbf {t}}_{\mathbf {k} = \mathbf {1}} \mathbf {\Sigma }_{\mathbf {b(k)}}$ where $var\left (\mathbf {u}_{\mathbf {b(k)}}\right) = \mathbf {\Sigma }_{\mathbf {b(k)}} = \mathbf {I} \sigma ^{2}_{\text {b(k)}}$. This means, *G*·*Y*, for example, is synonymous with **Z**_**b(1)**_ **u**_**b(1)**_, where **Z**_**b(1)**_ is the design matrix for genotype-by-year effects and $\mathbf {u}_{\mathbf {b}_{\mathbf {(1)}}}\phantom {\dot {i}\!}$ the vector of random genotype-by-year effects with $var\left (\mathbf {u}_{\mathbf {b(1)}}\right) = \mathbf {I}\sigma ^{2}_{\mathrm {b(1)}}$. The vector of errors associated with the records of **m** ^**(1)**^ is **ε**_**1**_ with *ε*_1_∼*N*(**0**,*R*_1_).

Finally, in the third stage, the GP model is implemented as

12 $$ \mathbf{m}^{\mathbf{(2)}} = \mathbf{1}\mathbf{\mu} + \mathbf{Z}_{\mathbf{g}} \mathbf{u}_{\mathbf{g}} + \mathbf{\epsilon}_{\mathbf{2}},   $$

where **m** ^**(2)**^ is the vector of adjusted genotype means across locations and years, **1** is a *m*×1 vector of ones, with *m* the number of genotypes, *μ* is the intercept, **Z**_**g**_ the marker matrix for genotypes, and **u**_**g**_ the vector of marker effects. We assume $\mathbf {u}_{\mathbf {g}} \sim N\left (\mathbf {0},\mathbf {I}\sigma ^{2}_{\mathrm {u}_{\mathrm {g}}}\right)$, thus $var\left (\mathbf {Z}_{\mathbf {g}} \mathbf {u}_{\mathbf {g}}\right) = \mathbf {Z}_{\mathbf {g}} \mathbf {Z}_{\mathbf {g}}^{\mathbf {T}}\sigma ^{2}_{\mathrm {u}_{\mathrm {g}}}$. The vector of errors is **ε**_**2**_ with **ε**_**2**_∼*N*(**0**,**R**_**2**_).

The difference between the two-stage (A2, and A3) and the three-stage (A4) approaches [using model (10) and model (12)] for GP-FV is the estimation of the *GY* effects, which in the first case makes use of the kinship matrix, whereas in the second case kinship is ignored.

#### Three-stage approach with kinship in the second stage: 3-stg-Kin (A5)

The three-stage approach can also make use of the kinship matrix in the second stage to dissect *GY* from *G* main effects.

The second-stage model is written as

13 $$ \mathbf{m}^{\mathbf{(1)}} = \mathbf{X} \mathbf{\beta} + \mathbf{Z}_{\mathbf{gy}} \mathbf{u}_{\mathbf{gy}} + \mathbf{Z}_{\mathbf{b}} \mathbf{u}_{\mathbf{b}} + \mathbf{\epsilon}_{\mathbf{1}},   $$

where **X** is the design matrix for fixed effects **β**. We keep *G* and *T* as fixed effects. **Z**_**b**_ is the design matrix and **u**_**b**_ is the vector of random effects between years for the random effects except the *GY* effects, for which we use **Z**_**gy**_ **u**_**gy**_, where **Z**_**gy**_ is the marker matrix for genotypes-by-year effects and **u**_**gy**_ is the vector of marker-by-year effects whose variance is $var\left (\mathbf {u}_{\mathbf {gy}}\right) = \mathbf {I}\sigma ^{2}_{\mathrm {u}_{\text {gy}}}$, such that $var(\mathbf {w}) = \mathbf {Z}_{\mathbf {gy}}\mathbf {Z}_{\mathbf {gy}}^{\mathbf {T}} \sigma ^{2}_{\mathrm {u}_{\text {gy}}}$. The vector of errors associated with the records of **m** ^**(1)**^ is **ε**_**1**_ with **ε**_**1**_∼*N*(**0**,**R**_**1**_). The third stage is the same as for the 3-stg-NoKin approach [model (12)] using the adjusted genotype means computed in the previous stage.

### Calculation of predictive ability - models for validation sets

Predictive abilities (*ρ*_*GP*_) were estimated as the Pearson correlation coefficient between the adjusted genotype means of the VS (**m** ^(2)^) and the GEBV $\left (\hat {\mathbf {v}} = \mathbf {Z}\hat {\mathbf {u}}\right)$. To estimate **m** ^(2)^ (adjusted genotype means) of the VS, we used a two-stage analysis, with model (3) as first stage to obtain adjusted genotype-tester means (**m** ^(1)^) across locations and years. In the second stage, the adjusted genotype means **m** ^(2)^ were estimated for VS_1_:GCA1-2012 and VS_3_:GCA1-2014 using the model

14 $$\begin{array}{*{20}l}  \mathbf{m}^{(1)} &= G + T : G \cdot T + L \cdot (G \times T) + \epsilon_{1}\\ &= G + T : G \cdot T + L + L \cdot G + L \cdot T + L \cdot G \cdot T\\ &\quad+ \epsilon_{1}, \end{array} $$

where all terms are defined as for model (1). For VS_2_:GCA1-2013, we did not include a location *L* main effect or a genotype-by-location effect *G*·*L* because testers and locations were totally confounded, thus the effect *L*·*T* represents *L*+*L*·*T* and *G*·*L*·*T* represents *G*·*L*+*G*·*L*·*T*. The model is

15 $$  \mathbf{m}^{(1)} = G + T : G \cdot T + L \cdot T + G \cdot T \cdot L + \epsilon_{1}.  $$

Adjusted genotype means based on models (14) and (15) (corresponding to VS_1_ and VS_3_, and VS_2_, respectively) are computed using best linear unbiased estimation (BLUE). Hence, predictive ability in each scenario was the Pearson correlation coefficient between the GEBV ($\hat {\mathbf {v}}$) from models (6), (7), (10) or (12) and **m** ^(2)^ of the VS from models (14) and (15), i.e.

16 $$  \rho_{GP} = corr\left(\hat{\mathbf{v}}, \mathbf{m}^{(2)}\right).  $$

## Results

### Structure of datasets and variance components

Variance components were estimated using the two-stage model (8) for all datasets (GER&PL, GER and PL), the three complete TS (TS_1_ [one cycle data], TS_2_ [two cycles data] and TS_3_ [three cycles data]) and the three VS (VS_1_:GCA1-2012, VS_2_:GCA1-2013 and VS_3_:GCA1-2014) (Table 2). The expected confounding of some effects due to the unbalancedness of the trials and the poor connectivity across cycles and between TS and VS is illustrated by the asymptotic correlation matrix for variance component estimates computed from the information matrix ([19], p. 248), e.g. for the GER&PL dataset TS_1_-VS_3_ (Additional file 1: Table S2 lower diagonal).

**Table 2:** Summary of variance component estimates in the three datasets

Dataset	TS	VS	*G*	*GY*	*L*	*GL*	*YL*	*GYL*	ac(*G* *L*,*G* *Y* *L*)
GER&PL	TS_1_	VS_1_	0.00	6.44	145.10	0.00	93.30	4.48	na
GER&PL	TS_1_	VS_2_	2.29	2.19	109.86	1.36	161.58	3.71	–0.89
GER&PL	TS_1_	VS_3_	6.45	2.72	166.48	2.41	117.31	5.08	–0.92
GER	TS_1_	VS_1_	6.75	0.58	143.57	1.11	92.65	3.83	–0.89
GER	TS_1_	VS_2_	3.74	1.04	113.46	1.08	169.73	4.03	–0.88
GER	TS_1_	VS_3_	4.55	0.93	173.53	1.41	108.66	4.68	–0.92
PL	TS_1_	VS_1_	0.00	5.68	160.05	0.00	85.39	4.28	na
PL	TS_1_	VS_2_	0.00	3.41	108.72	1.72	155.03	3.03	–0.90
PL	TS_1_	VS_3_	0.00	11.28	173.99	3.24	94.82	5.17	–0.98
GER&PL	TS_2_	VS_1_	5.85	1.77	132.51	0.80	89.24	3.17	–0.96
GER&PL	TS_2_	VS_2_	4.18	1.54	110.06	1.27	149.52	2.78	–0.96
GER&PL	TS_2_	VS_3_	7.42	1.56	166.22	1.60	108.97	3.92	–0.97
GER	TS_2_	VS_1_	8.00	0.29	142.97	1.15	89.21	3.06	–0.93
GER	TS_2_	VS_2_	5.98	0.44	112.15	1.49	161.93	2.92	–0.94
GER	TS_2_	VS_3_	6.89	0.13	172.96	1.62	109.00	3.44	–0.93
PL	TS_2_	VS_1_	0.00	6.12	135.17	0.00	84.60	4.17	na
PL	TS_2_	VS_2_	0.00	4.22	89.73	0.004	155.83	4.00	–0.97
PL	TS_2_	VS_3_	0.00	9.97	158.31	0.00	92.84	6.13	na
GER&PL	TS_3_	VS_1_	2.24	4.53	163.69	0.68	86.92	3.89	–0.87
GER&PL	TS_3_	VS_2_	5.09	1.51	159.44	1.11	93.36	4.07	–0.81
GER&PL	TS_3_	VS_3_	7.32	1.02	176.06	1.18	85.59	4.84	–0.86
GER	TS_3_	VS_1_	7.19	1.10	170.60	0.78	86.35	3.66	–0.80
GER	TS_3_	VS_2_	7.02	0.38	186.59	1.18	84.42	4.14	–0.80
GER	TS_3_	VS_3_	7.01	0.32	166.34	1.16	88.32	3.69	–0.76
PL	TS_3_	VS_1_	0.00	5.33	156.13	0.77	84.70	3.80	–0.94
PL	TS_3_	VS_2_	0.67	5.00	144.19	0.97	93.47	4.10	–0.85
PL	TS_3_	VS_3_	5.19	3.61	161.72	0.99	81.25	5.20	–0.90

German and Polish together (GER&PL), only German (GE) and only Polish (PL), for all the training set (TS) and validation set (VS) combinations. Reported effects use the factors: Genotypes (*G*), year (*Y*) and location (*L*). ac(*G* *L*,*G* *Y* *L*) is the asymptotic correlation between variance component estimates of *GL* and *GYL* effects. na represents non-estimable values due to a zero value of a variance component

TS_1_: GCA1-2009 + GCA2-2010 + GCA3-2011, TS_2_: GCA1-2009 + GCA2-2010 + GCA1-2010 + GCA2-2011, TS_3_: GCA1-2009 + GCA2-2010 + GCA3-2011 + GCA1-2010 + GCA2-2011 + GCA3-2012 + GCA1-2011 + GCA2-2012 + GCA3-2013, VS_1_: GCA1-2012, VS_2_: GCA1-2013, VS_3_: GCA1-2014

The correlation between variance component estimates for *G* and *GY* is −0.8747, for *L* and *YL* it is −0.2556, for *GL* and *GYL* it is −0.9229, for *GTL* and *GTYL* it is −0.9758 and between *GT* and *GTY* it is −0.9491. The confounding is also observed in the asymptotic correlation matrix for variance component estimates of the TS_1_ scenarios (Additional file 1: Tables S3 and S4). For the TS_2_ (Additional file 1: Tables S5–S7) and the TS_3_ (Additional file 1: Tables S8–S10) scenarios, the confounding is still visible, though in rather lower magnitudes.

An asymptotic correlation of ≃−1 indicates ill-conditioning ([20], p156). Confounding of effects is the limiting case of ill-conditioning when the asymptotic correlation between two effects is exactly −1. It is clear that the extreme unbalancedness of the datasets renders variance component estimates unstable, in the sense that a few genotypes in the analysis impact strongly on the relative contribution of each effect to the total variance.

Additionally, variance components for genotype main effects (*G*) in the PL dataset are most of the times estimated as zero as well as for *GL* interaction effects, reflecting the poor connectivity of the datasets. The asymptotic correlations between the variance component estimates of *GL* and genotype-by-year-by-location interaction (*GYL*) effects were marginally more negative for the Polish scenarios than for the German ones (Table 2). This could be due to a different trial allocation across years and locations in Poland than in Germany. The GER dataset has more locations per year that are not repeated across the other years, whereas in the PL dataset fewer locations are used across years, that is, more locations are repeated across years, i.e., the number of location-year combinations compared to the number of total locations across years are greater in the GER than in the PL datasets (Additional file 1: Table S1). This situation reflects more confounding for the PL dataset, and as a consequence, the PL dataset does not have as many *GL* or *GYL* effects as the GER dataset, so that asymptotic correlations between the variance estimates for *GL* and *GYL* effects are slightly higher in absolute value for the PL program than for the GER program (Table 2). The confounding is diminished when more years are used in the TS because the number of year-location combinations increases.

### Predictive abilities

**Fig. 2:**
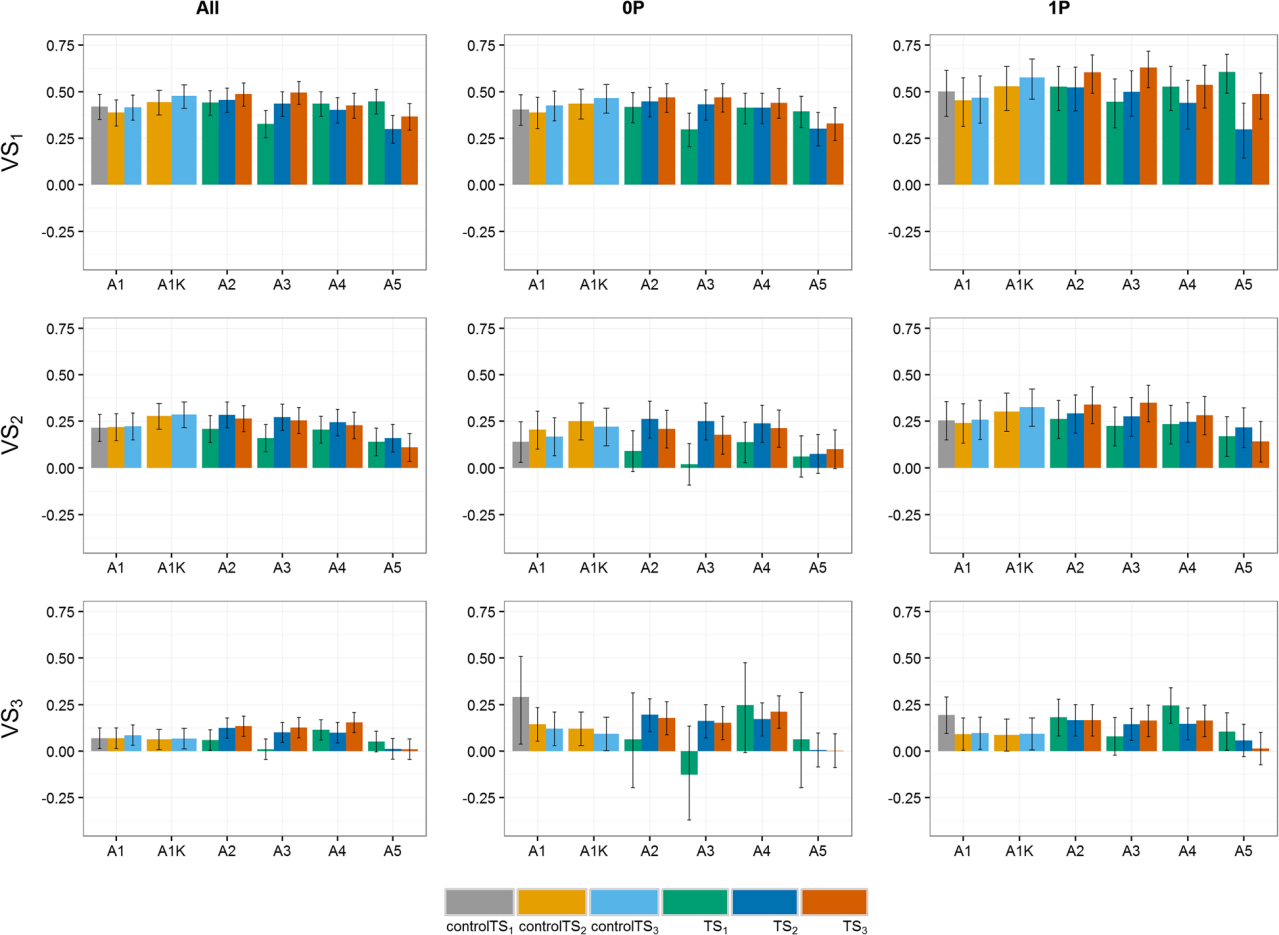
Predictive abilities (y-axis) of the **German and Polish** dataset for the three scenarios. TS_1_ and controlTS_1_, TS_2_ and controlTS_2_, and TS_3_ and controlTS_3_ to predict the validation sets VS_1_, VS_2_ and VS_3_ with All, 0P and 1P-scenarios. *Black lines* for each bar represent the 95% confidence intervals of the predictive ability. Year-wise approach (A1) and year-wise with kinship approach (A1K) were fitted to the control sets, approaches 2-stg-Kin (A2), 2-stg-Kin-het (A3), 3-stg-NoKin (A4) and 3-stg-Kin (A5) to the complete sets. TS_1_: GCA1-2009 + GCA2-2010 + GCA3-2011, controlTS_1_: GCA1-2009, TS_2_: GCA1-2009 + GCA2-2010 + GCA1-2010 + GCA2-2011, controlTS_2_: GCA1-2009 + GCA1-2010, TS_3_: GCA1-2009 + GCA2-2010 + GCA3-2011 + GCA1-2010 + GCA2-2011 + GCA3-2012 + GCA1-2011 + GCA2-2012 + GCA3-2013, controlTS_3_: GCA1-2009 + GCA1-2010 + GCA1-2011, VS_1_: GCA1-2012, VS_2_: GCA1-2013, VS_3_: GCA1-2014

**Fig. 3:**
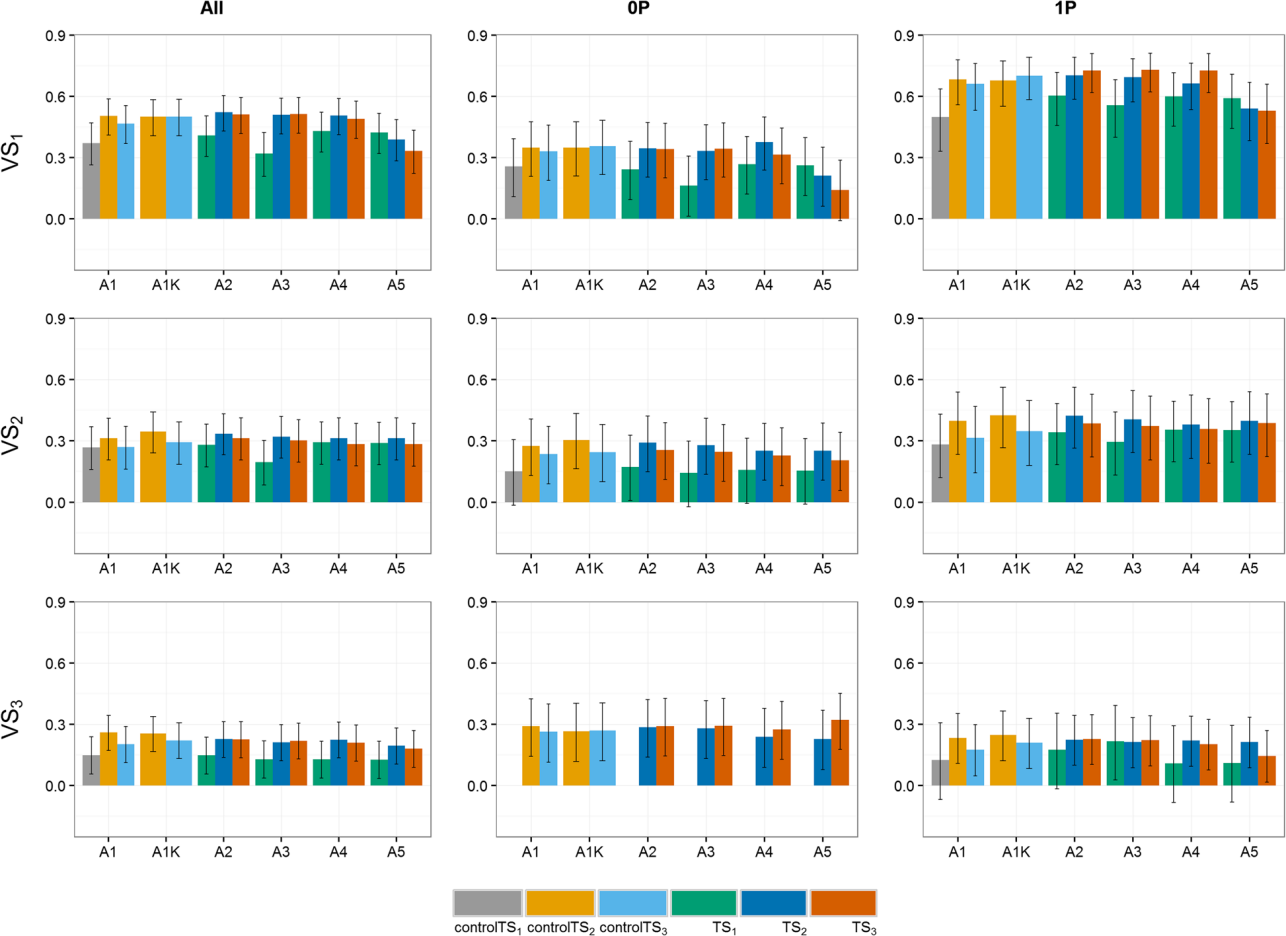
Predictive abilities (y-axis) of the **German** dataset for the three scenarios. TS_1_ and controlTS_1_, TS_2_ and controlTS_2_, and TS_3_ and controlTS_3_ to predict the validation sets VS_1_, VS_2_ and VS_3_ with All-, 0P- and 1P-scenarios. *Black lines* for each bar represent the 95% confidence intervals of the predictive ability. Year-wise approach (A1) and year-wise with kinship approach (A1K) were fitted to the control sets, approaches 2-stg-Kin (A2), 2-stg-Kin-het (A3), 3-stg-NoKin (A4) and 3-stg-Kin (A5) to the complete sets. TS_1_: GCA1-2009 + GCA2-2010 + GCA3-2011, controlTS_1_: GCA1-2009, TS_2_: GCA1-2009 + GCA2-2010 + GCA1-2010 + GCA2-2011, controlTS_2_: GCA1-2009 + GCA1-2010, TS_3_: GCA1-2009 + GCA2-2010 + GCA3-2011 + GCA1-2010 + GCA2-2011 + GCA3-2012 + GCA1-2011 + GCA2-2012 + GCA3-2013, controlTS_3_: GCA1-2009 + GCA1-2010 + GCA1-2011, VS_1_: GCA1-2012, VS_2_: GCA1-2013, VS_3_: GCA1-2014

**Fig. 4:**
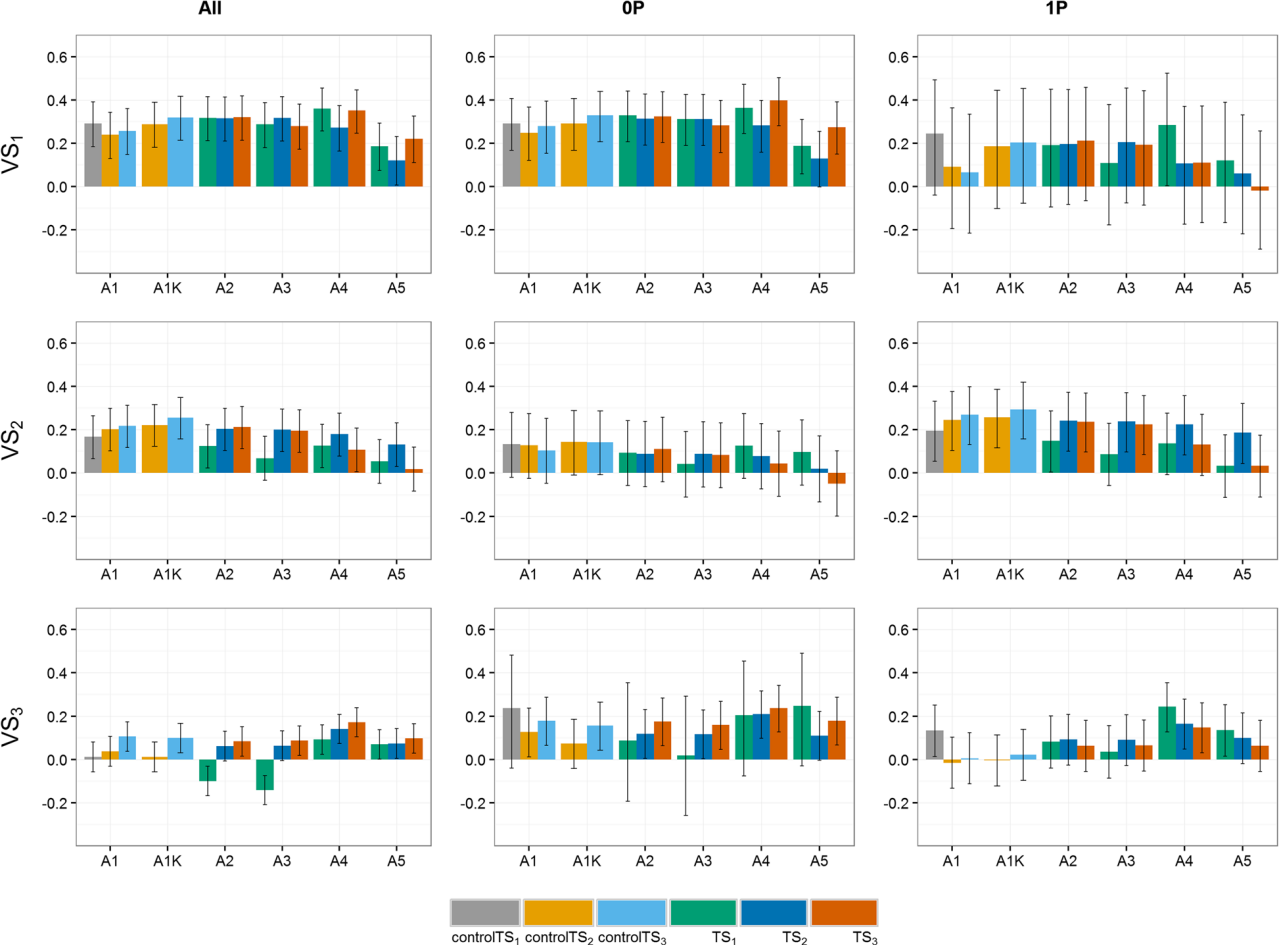
Predictive abilities (y-axis) of the **Polish** dataset for the three scenarios. TS_1_ and controlTS_1_, TS_2_ and controlTS_2_, and TS_3_ and controlTS_3_ to predict the validation sets VS_1_, VS_2_ and VS_3_ with All-, 0P- and 1P-scenarios. *Black lines* for each bar represent the 95% confidence intervals of the predictive ability. Year-wise approach (A1) and year-wise with kinship approach (A1K) were fitted to the control sets, approaches 2-stg-Kin (A2), 2-stg-Kin-het (A3), 3-stg-NoKin (A4) and 3-stg-Kin (A5) to the complete sets. TS_1_: GCA1-2009 + GCA2-2010 + GCA3-2011, controlTS_1_: GCA1-2009, TS_2_: GCA1-2009 + GCA2-2010 + GCA1-2010 + GCA2-2011, controlTS_2_: GCA1-2009 + GCA1-2010, TS_3_: GCA1-2009 + GCA2-2010 + GCA3-2011 + GCA1-2010 + GCA2-2011 + GCA3-2012 + GCA1-2011 + GCA2-2012 + GCA3-2013, controlTS_3_: GCA1-2009 + GCA1-2010 + GCA1-2011, VS_1_: GCA1-2012, VS_2_: GCA1-2013, VS_3_: GCA1-2014

Predictive abilities were calculated using Eq. (16) (Figs. 2, 3 and 4). Notice that the year-wise with kinship approach (A1K) is not used for controlTS_1_ because the control TS is composed of only one year, thus fitting a *GY* effect would over-parametrize the model.

There are years or cycles that are easier to predict than others. Predicting the VS_1_:GCA1-2012 had, across all datasets, the highest predictive abilities. VS_2_:GCA1-2013 had also relatively high *ρ*_*GP*_ compared to VS_3_:GCA1-2014.

There was a marginal increase in *ρ*_*GP*_ along the approaches from TS covering data from two and three selection cycles (TS_2_ and TS_3_) over TS_1_ (one selection cycle). In the GER&PL program, this increase is observed especially for VS_1_ and for the 1P-scenario of VS_2_. In Germany the difference between TS_2_ and TS_3_ is small, though there is a general increase of the predictive ability in these two datasets over TS_1_. In the PL dataset, *ρ*_*GP*_ obtained using TS_3_ or TS_2_ are not always better than TS_1_. They depend on the model and the VS used.

When relatedness between TS and VS increased, there was a general increase in *ρ*_*GP*_. The increment depends on the dataset, the target VS and the model (particularly for the PL dataset). For example, in the VS_1_ of the GER dataset, the increase in *ρ*_*GP*_ from the 0P- to the 1P-scenario was from ∼ 0.30 to ∼ 0.60, and in the pooled dataset (GER&PL) from ∼ 0.40 to ∼ 0.50, whereas in the PL dataset the 1P-scenario had too wide confidence intervals and varying predictive abilities across models, so that no general trend can be recognized. For VS_3_, there was no increase in *ρ*_*GP*_ from the 0P- to the 1P-scenario. This is in agreement with the Euclidean distances presented in Additional file 1: Table S11.

**Fig. 5:**
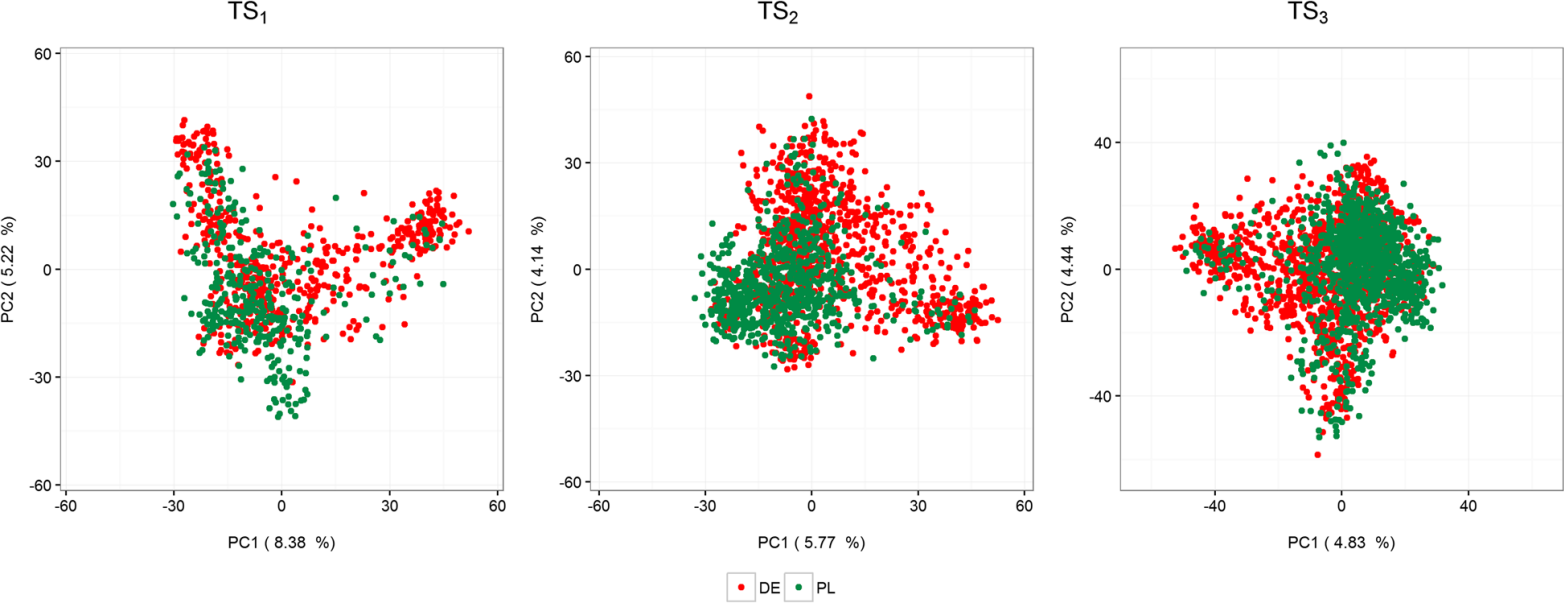
Principal component (PC) plots for the training datasets TS_1_, TS_2_ and TS_3_ of the German (GER) and the Polish (PL) programs. TS_1_: GCA1-2009 + GCA2-2010 + GCA3-2011, TS_2_: GCA1-2009 + GCA2-2010 + GCA1-2010 + GCA2-2011, TS_3_: GCA1-2009 + GCA2-2010 + GCA3-2011 + GCA1-2010 + GCA2-2011 + GCA3-2012 + GCA1-2011 + GCA2-2012 + GCA3-2013

Predictive abilities were on average higher for the GER dataset (0.2741) than for the GER&PL program (0.2407) and markedly higher than for the PL dataset (0.1424). When splitting German and Polish genotypes within the GER&PL dataset, *ρ*_*GP*_ for only Polish lines was lower than the *ρ*_*GP*_ obtained when only considering the PL program, whereas the *ρ*_*GP*_ obtained for German lines within the GER&PL dataset was higher than that obtained from the GER dataset alone. The principal component analysis (PCA) of the marker data in Fig. 5 shows that the genotypes from the PL program form a more compact cloud than those from the GER program and that the Polish lines are well contained within the cloud of the German lines. Although the first two principal components capture little variance (<15*%*), the PCA shows that lines in the PL program are more closely related than lines in the GER program, so that some far related German lines could cause a bias in the prediction of the Polish lines within the GER&PL dataset.

For controlTS_2_ and controlTS_3_, approach A1K (year-wise with kinship) was on average 17% higher in predictive ability than A1 (year-wise without kinship) across programs, relatedness scenarios, TS and VS (17.3% in the GER dataset, 21.8% in the PL dataset and 13.0% in the GER&PL dataset). Approaches A2 (2-stg-Kin), A3 (2-stg-Kin-het) and A4 (3-stg-NoKin) yielded very similar predictive abilities across datasets, relatedness scenarios and VS for TS_2_ and TS_3_ (on average 0.2497), and were also very close to predictive abilities obtained by A1K (on average 0.2477). The worst approach was A5 (3-stg-Kin), which led on average to 23% lower *ρ*_*GP*_ than the average of A2, A3 and A4 across programs, relatedness scenarios and VS.

#### Predictive abilities in sampling scenarios

To avoid the confounding effect of the VS-size and to objectively compare parent scenarios and models, we defined a VS-size of 100 genotypes to be predicted and iterated the GP-FV 10 times. Results are depicted in Additional file 1: Figures S5–S7. The predictive abilities and their 95% confidence intervals are based on the mean of the 10 sample draws.

The predictive abilities obtained for the scenarios with samples of 100 genotypes in the VS confirmed the trends observed for scenarios with complete validation sets (Figs. 2, 3 and 4). The size of the confidence intervals varied between the sampling scenarios and the scenarios using all available genotypes. For smaller VS-size (sampling 100 genotypes), confidence intervals are wider, suggesting that more and better data would allow better genotype estimates, as expected.

### Relatedness scenarios

A PCA for each combination TS-VS-relatedness scenario in all the datasets (GER&PL, GER and PL) showed that PC1 and PC2 captured only little variance (<15*%*) (Additional file 1: Figures S8–S16), but still showed that TS and VS are genetically structured and there is no clear separation for TS and VS using different relatedness degrees, i.e., different parent number in the TS.

Additionally, the mean of the Euclidean distance using the marker matrix for genotypes in TS and all relatedness scenarios of VS (Additional file 1: Table S11), showed no strong variation between relatedness scenarios and between TS-VS combinations. The values were in general slightly higher for the PL dataset than for the GER dataset, showing that the two groups are closely related within themselves but marginally genetically divergent between them. The results are consistent with the PCAs, since there was no clear pattern from the 1P-scenarios that would suggest a closer relatedness between TS and VS than the 0P-scenarios or the All-scenarios.

For the three relatedness scenarios (All, 0P- and 1P-scenarios) across all the datasets (GER, PL and GER&PL), approach A1K (year-wise with kinship) produced, in general, very similar predictive abilities to approaches A2 (2-stg-Kin), A3 (2-stg-Kin-het) and A4 (3-stg-NoKin), and these four approaches were on average 18% better than approaches A1 (year-wise without kinship) and A5 (3-stg-Kin) in terms of *ρ*_*GP*_. In the GER and GER&PL datasets, A1K produced slightly higher predictive abilities than A2, A3 and A4 for All- and 0P-scenarios, whereas for 1P-scenario there was no markedly difference between A1K and A2, A3 and A4. In the PL program, A4 had on average 13% and 8% higher *ρ*_*GP*_ than A1K for the 0P- and 1P-scenario, respectively. For the All-scenario, A4 showed no difference with A1K and both approaches yielded on average 14% better *ρ*_*GP*_ than A2 and A3.

### Top-yield scenarios

**Fig. 6:**
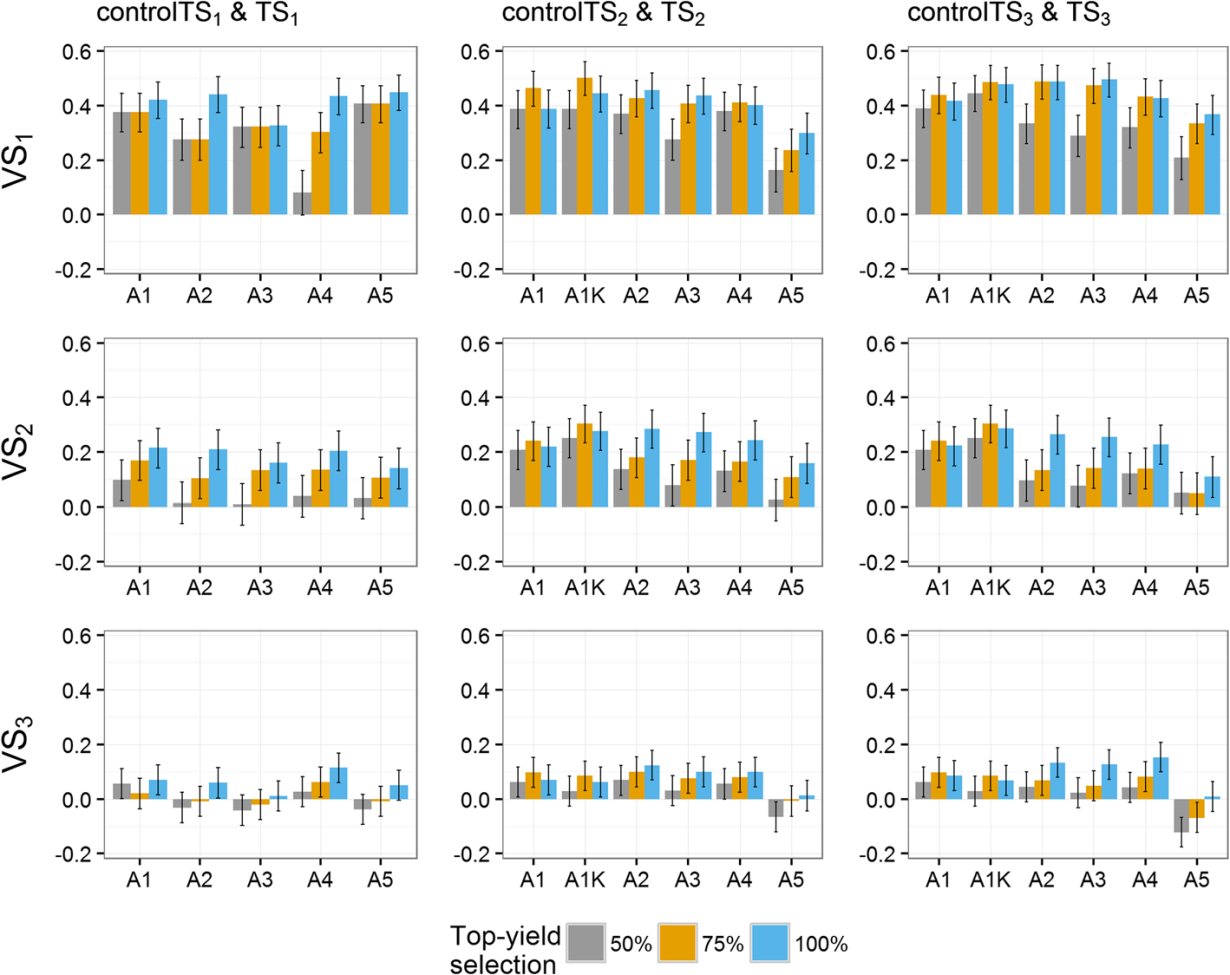
Predictive abilities (y-axis) of the **German and Polish** dataset for selection scenarios of top-yield performance. Selection in the training set (TS): 50% of highest yielding genotypes (*gray bars*), 75% of highest yielding genotypes (*yellow bars*) and 100% of the genotypes (*blue bars*), using validation sets VS_1_, VS_2_ and VS_3_. *Black lines* for each bar represent the 95% confidence intervals of the predictive ability. Year-wise approach (A1) and year-wise with kinship approach (A1K) were fitted to the control TS, approaches 2-stg-Kin (A2), 2-stg-Kin-het (A3), 3-stg-NoKin (A4) and 3-stg-Kin (A5) to the complete TS. TS_1_: GCA1-2009 + GCA2-2010 + GCA3-2011, controlTS_1_: GCA1-2009, TS_2_: GCA1-2009 + GCA2-2010 + GCA1-2010 + GCA2-2011, controlTS_2_: GCA1-2009 + GCA1-2010, TS_3_: GCA1-2009 + GCA2-2010 + GCA3-2011 + GCA1-2010 + GCA2-2011 + GCA3-2012 + GCA1-2011 + GCA2-2012 + GCA3-2013, controlTS_3_: GCA1-2009 + GCA1-2010 + GCA1-2011, VS_1_: GCA1-2012, VS_2_: GCA1-2013, VS_3_: GCA1-2014

**Fig. 7:**
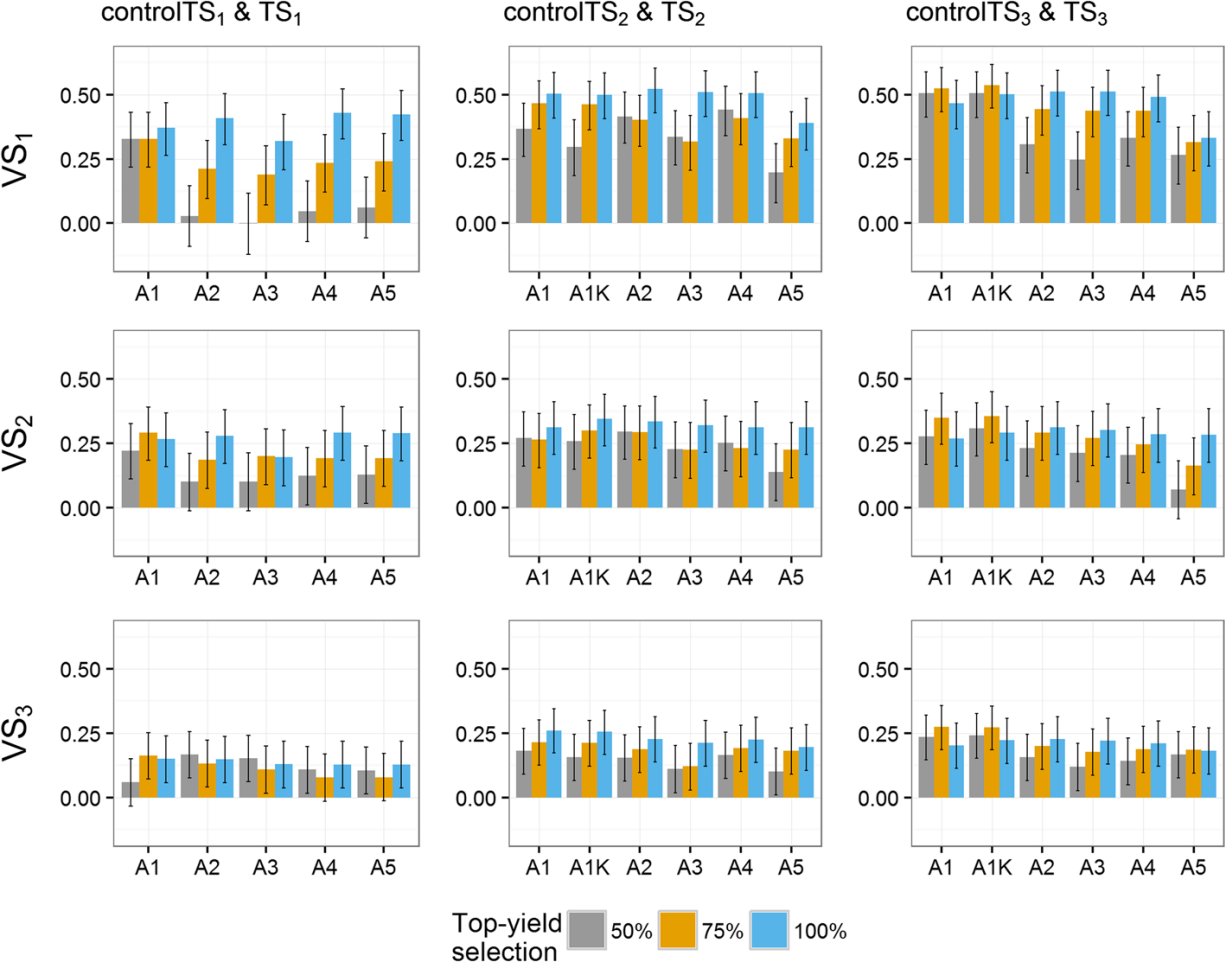
Predictive abilities (y-axis) of the **German** dataset for selection scenarios of top-yield performance. Selection in the training set (TS): 50% of highest yielding genotypes (*gray bars*), 75% of highest yielding genotypes (*yellow bars*) and 100% of the genotypes (*blue bars*), using validation sets VS_1_, VS_2_ and VS_3_. *Black lines* for each bar represent the 95% confidence intervals of the predictive ability. Year-wise approach (A1) and year-wise with kinship approach (A1K) were fitted to the control TS, approaches 2-stg-Kin (A2), 2-stg-Kin-het (A3), 3-stg-NoKin (A4) and 3-stg-Kin (A5) to the complete TS. TS_1_: GCA1-2009 + GCA2-2010 + GCA3-2011, controlTS_1_: GCA1-2009, TS_2_: GCA1-2009 + GCA2-2010 + GCA1-2010 + GCA2-2011, controlTS_2_: GCA1-2009 + GCA1-2010, TS_3_: GCA1-2009 + GCA2-2010 + GCA3-2011 + GCA1-2010 + GCA2-2011 + GCA3-2012 + GCA1-2011 + GCA2-2012 + GCA3-2013, controlTS_3_: GCA1-2009 + GCA1-2010 + GCA1-2011, VS_1_: GCA1-2012, VS_2_: GCA1-2013, VS_3_: GCA1-2014

**Fig. 8:**
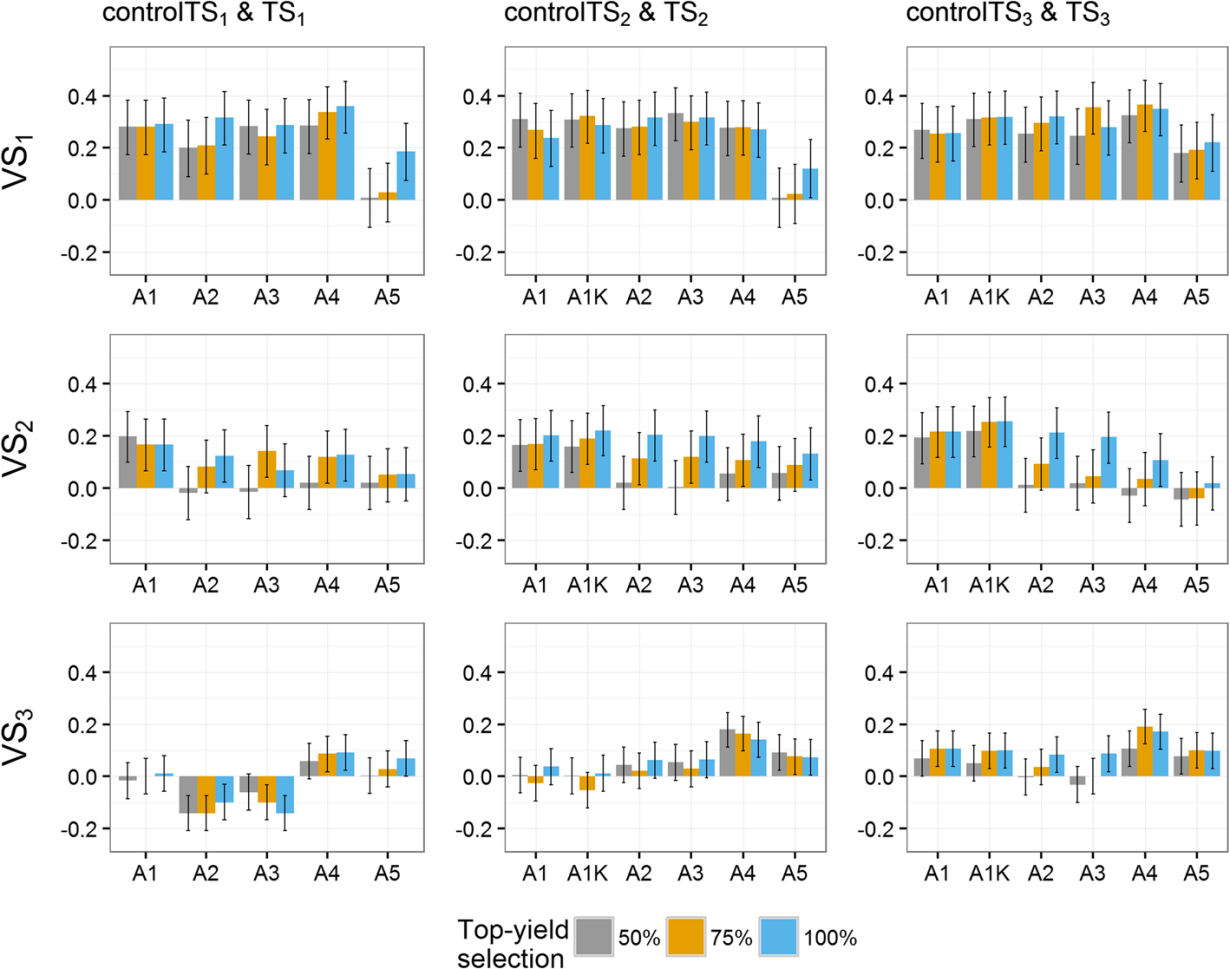
Predictive abilities (y-axis) of the **Polish** dataset for selection scenarios of top-yield performance. Selection in the training set (TS): 50% of highest yielding genotypes (*gray bars*), 75% of highest yielding genotypes (*yellow bars*) and 100% of the genotypes (*blue bars*), using validation sets VS_1_, VS_2_ and VS_3_. *Black lines* for each bar represent the 95% confidence intervals of the predictive ability. Year-wise approach (A1) and year-wise with kinship approach (A1K) were fitted to the control TS, approaches 2-stg-Kin (A2), 2-stg-Kin-het (A3), 3-stg-NoKin (A4) and 3-stg-Kin (A5) to the complete TS. TS_1_: GCA1-2009 + GCA2-2010 + GCA3-2011, controlTS_1_: GCA1-2009, TS_2_: GCA1-2009 + GCA2-2010 + GCA1-2010 + GCA2-2011, controlTS_2_: GCA1-2009 + GCA1-2010, TS_3_: GCA1-2009 + GCA2-2010 + GCA3-2011 + GCA1-2010 + GCA2-2011 + GCA3-2012 + GCA1-2011 + GCA2-2012 + GCA3-2013, controlTS_3_: GCA1-2009 + GCA1-2010 + GCA1-2011, VS_1_: GCA1-2012, VS_2_: GCA1-2013, VS_3_: GCA1-2014

In the present study, using a selected fraction of individuals in the TS was useful only in the control TS, i.e., when a given selection cycle (genetic background) was represented by only one year of (GCA1) data (Figs. 6, 7 and 8). In this case, the effects of non-yield QTL are confounded within each genetic background with the *GY* effects. Consequently, a selected fraction of individuals with higher grain yield performance will reduce variation due to non-yield QTL and, therefore, reduce bias due to confounding effects. In contrast, when two or more years of data are available per genetic background, environmental and non-yield QTL effects can be estimated separately, thus rendering the use of selected fractions in the TS (Top75% or Top50%) non-effective.

For the control TS across all datasets, the Top75% and Top100% scenarios using the year-wise (A1) approach and year-wise with kinship (A1K) approach had a higher *ρ*_*GP*_ than the Top50% scenario. For the GER and GER&PL datasets A1K using Top75% was marginally better than A1K using Top100% (on average 4% better) and across all datasets, A1K had 13% higher *ρ*_*GP*_ than A1. Additionally, for A2 (2-stg-Kin), A3 (2-stg-Kin-het), A4 (3-stg-NoKin) and A5 (3-stg-Kin) the Top100% scenarios outperformed the Top75% and Top50% scenarios in terms of *ρ*_*GP*_.

## Discussion

The key contribution of this paper was an evaluation of the use of kinship to model *GY* effects in disconnected datasets for a better separation from GEBV. We presented a detailed step-by-step genomic prediction analysis modeling *GY* with different approaches and extending the use of molecular markers to deal with disconnected trials. We also use a validation set system across years that approximates to the breeders’ aim of empirical validation.

In the analyzed datasets, we found that *G* and *GY* effects (and other effects that include factor *G*) were confounded. This was evident from the large negative asymptotic correlations that reflect ill-conditioning (Tables 2, Additional file 1: Tables S2–S4). Using multiple genetic backgrounds as in TS_2_ (two selection cycles) and TS_3_ (three selection cycles), it is possible in principle to build bridges across years given that *GY* is specific to the genetic background. Nonetheless, the unbalancedness of the design was still so strong that those effects remained confounded (Additional file 1: Tables S5–S10). The use of several cycles improved the estimate of the variance of genotype effects because there were more lines repeated across years within cycles (especially in the PL dataset), thus solving the problem of a zero variance estimate with single-cycle data. By contrast, the use of multiple cycles did not solve the ill-conditioning problem.

The main advantage expected from pooling GCA1+GCA2+GCA3 data in the TS is that a better bridge is built between years, leading to more precise adjusted means, thus allowing to dissect *GY* from GEBV. If most of the interaction is specific to the genetic background (as we assume it to be), multiple genetic backgrounds (selection cycles) are needed for a better separation of main SNP effects, such as in TS_2_ and TS_3_. Auinger et al. [4] recently found that aggregating data from several consecutive cycles connected by a sufficient number of common ancestors improves the accuracy of the predictions of candidate genotypes. Our results confirm their conclusion and complement the recommendation towards using additionally a selected fraction of 75% best yielding genotypes in the TS to reduce biasing effects due to non-yield QTL. The most surprising result is that the highest and most stable results are obtained with the controlTS_2_ and controlTS_3_ with A1K, i.e., using GCA2 and GCA3 data apparently is not only advantageous, but leads to a slight reduction in prediction abilities in comparison to using multiple consecutive GCA1 data, as in A1K. This is probably due to a biased segregation and variation of QTL effects in the selected fractions of GCA2 and GCA3 with respect to the non-selected GCA1 datasets.

The advantage of using a whole cycle with GCA1 to GCA3 is that the genotypes making it to GCA2 and GCA3 have been seen in more than one year, thus models that use a complete TS benefit from the TS structure, allowing reasonable *GY* estimates with or without kinship. By using only GCA1 experiments (i.e., control TS), a good coverage of the genetic target population is achieved and the use of kinship to model the genetic connection across years (specifically with model A1K) seems to be powerful enough to estimate *GY* fairly independent from GEBVs. Modeling *GY* is essential when there is no connectivity between years as different sets of genotypes are tested each year. By contrast, there is excellent connectivity between locations in each year through genotypes and checks because the same set of genotypes is usually tested at all or most locations. Thus, we expect that the *GL* effect estimates are relatively accurate within a year whereas modeling *GY* is the Achilles’ heel of the whole analysis as *GY* will be confounded with the GEBVs.

The PL dataset produced markedly lower predictive abilities than the GER and the GER&PL datasets. We had stated that the German genotypes profited from the Polish ones but not *vice versa*, perhaps because the GER program is genetically more diverse than the PL program (Fig. 5), so that there are some SNPs that are monomorphic for the Polish lines but not for the German lines causing a bias in the prediction of the Polish lines within the GER&PL dataset. Probably the main reason why the PL dataset had markedly lower predictive abilities than the GER dataset is that the Polish data have a higher error, i.e., *GY*, *GL* and *GYL* interaction effects are estimated less accurately. The fact that in Poland there are fewer *GL* and *GYL* evaluations (Additional file 1: Table S1) could explain why the Polish predictive abilities were lower. Endelman et al. [21] show that having larger populations spread across more environments produces higher predictive abilities than evaluating the same genotypes in fewer environments. The GER dataset has a higher number of *GL* and *GYL* combinations because trials with Tester 1 and Tester 2 are not evaluated in exactly the same locations, whereas in the PL dataset, there is a balanced design of testers across locations within a year.

Predictive abilities were in general 26% higher for the 1P-scenarios than the 0P-scenarios and 15% higher than for the All-scenarios, reinforcing the findings of other genomic prediction studies on the effect of relationships between TS and VS [22–26]. The use of the kinship to model *GY* in 0P-scenarios did not consistently compensate the lack of relatedness. Although the three relatedness scenarios (All-, 0P- and 1P-scenarios) showed small differentiation by the mean Euclidean distance (Additional file 1: Table S11) and not so marked divergence in the PCA plots (Additional file 1: Figures S8–S16), a realized relationship between TS and VS does have a positive impact on the predictive abilities. In the best case, i.e. the GER dataset - VS_1_:GCA1-2012, predictive abilities ranged from ∼0.14 to ∼0.38 in the 0P-scenario and from ∼0.50 to ∼0.73 in the 1P-scenario.

All approaches revealed marked variation in predictive abilities across scenarios. In general, there was a modest increment of the year-wise with kinship approach (A1K) over the year-wise approach (A1), in particular controlTS_2_:GCA1-2009 + GCA1-2010 and controlTS_3_:GCA1-2009 + GCA1-2010 + GCA1-2011 over controlTS_1_:GCA1-2009. The confidence intervals of the predictive abilities of the year-wise approach (A1) overlapped most of the times with predictive abilities of the year-wise with kinship approach (A1K) (black lines of Figs. 2, 3 and 4), but even so, in challenging programs as the Polish one, the benefit of using the kinship was worth about 22% on the correlation scale. In the GER and GER&PL datasets the approaches A2, A3 and A4 had consistent and very similar predictive abilities. Only A5 was almost always markedly lower in predictive ability than the other models. From these results we conclude first, that using the kinship to model *GY* for settings of disconnected years is safer than estimating the year effect as the simple average of the genotypes evaluated in a given year, and second, when the datasets cover multiple genetic backgrounds in the same year (as datasets used for A2, A3, A4 and A5), it is possible to estimate *GY* effects either by using kinship directly in the GP stage (A2 and A3) or simply using the correct model in the TS to obtain adjusted genotype means across years (A1K) and submit them to GP. Hence, kinship is helpful in the case of disconnected data and no harm is done using it in other cases. Although computational load may increase with the use of kinship to model *GY*, novel approaches that combine dense and sparse matrix methods alleviate this burden and are starting to become freely available [27].

It was surprising that the 3-stg-Kin approach (A5) had markedly lower predictive abilities than the approach 3-stg-NoKin (A4) because the difference between both approaches is that in A5, we use kinship to model the *GY* whereas in A4 we do not, so we would have expected that using kinship in modeling *GY* improves predictive ability. While this expectation was confirmed in the other approaches that used kinship (A2 and A3), this was not the case here. All methods are designed to approximate the same single-stage model (1), so that it was not obvious which one should work better because it uses kinship to model the *GY* effects, as does model (1). While both A5 and A1K seek to approximate the single-stage model (1), A1K makes somewhat weaker assumptions because it does not use kinship to model *GY* in the second stage. So while A5 better approximates the single-stage model, there is no guarantee that the single-stage model is the best model for GP. This may explain why A1K does better in terms of predictive accuracy and also why A4 fared better than A5.

Predictive abilities for VS_1_:GCA1-2012 ranged from ∼0.24 on average in the PL dataset to maximum ∼0.73 in the GER dataset, and the lowest *ρ*_*GP*_ occurred for VS_3_:GCA1-2014 ranging from zero (or negative) in the worst case of the PL dataset to ∼0.33 the best case in the GER dataset. The results that we obtained are in accordance with the predictive abilities reported by Auinger et al. [4], which ranged between 0.39 and 0.58 (with an average heritability of 0.83) and were based on GP-FV. The validation sets VS_1_:GCA1-2012 and VS_2_:GCA1-2013 could be predicted more accurately than VS_3_:GCA1-2014. Data from the year 2014 has been identified as problematic, since it is not easy to predict within the GP program from KWS-LOCHOW. We acknowledge the fact that the scenarios TS_3_-VS_1_ and TS_3_-VS_2_ are less realistic in the sense that data from the same year of prediction is used in TS and VS, but we consider those scenarios because the number of genotypes in GCA3-2012 and GCA3-2013 is low (less than 30 shared genotypes within cycles in all the programs) and there are no genotypes in common between TS and VS, keeping our condition of disconnected TS and VS valid for the presented scenarios. Moreover, removing data from GCA3-2012 or GCA3-2013 from TS_3_ led to only a slight variation in the value of the reported predictive abilities, with changes occurring after the third of fourth decimal place.

Besides focusing in the mean performance across years, another important target in plant breeding is to investigate stability, which refers to the variability from year to year. In the context of genomic prediction, it makes sense to also study the expected consistency of year to year performance aiming to minimize this variability [8, 28]. This stability aspect deserves further study.

The results obtained for the top-yield scenarios led us to conclude that using a multiple genetic background in the TS allows capturing the true QTL for yield, whereas when having only one year in the TS (i.e. control sets), the model is not able to do this distinction and hence, a pre-selection of best yielding genotypes may improve the predictive abilities. This explains the ability of the year-wise with kinship approach (A1K) to improve *ρ*_*GP*_ using 75% of the best-yielding genotypes even if the TS-size was reduced. Selecting a top fraction of best yielding genotypes for the TS basically allows to reduce the genotype-by-year effects that cannot be accurately estimated due to absence of connectivity across years. In this work, we randomly used 75% top fraction, but other values (e.g 95%, 90%, 85%, 80%) should be further considered. The implementation of the A1K (year-wise with kinship) approach is advantageous from the technical point of view, since the analysis requires lower computing power than using 100% data from complete cycles as for A2, A3 and A4. Given the overlap of the 95% confidence intervals for the majority of the approaches across scenarios, there is no single method that always outperforms the contending methods. Nevertheless, our favorite approach for GP using disconnected years of a breeding program with a similar structure to the one described in the present work is the year-wise with kinship (A1K) approach with TS composed of minimum two single years of multiple genetic backgrounds (i.e. controlTS_2_ and controlTS_3_). Our reasons for this preference are as follows. For the A1K approach the predictive abilities were more stable across scenarios, including that the relationship information (kinship matrix) ensured that *GY* were properly estimated, computing load was manageable and a pre-selection of the best genotypes in the TS did not have negative effects over the predictive abilities.

## Conclusions

The main conclusions of this study are: (i) Using multi-year datasets is advantageous, (ii) the year-wise with kinship approach (A1K) with two or three years in the TS (controlTS_2_ or controlTS_3_) was our favourite since it led to slightly better and more consistent *ρ*_*GP*_ trend than any other approach, (iii) the use of kinship to model *GY* in multi-year datasets is encouraged, especially for datasets covering multiple genetic backgrounds and where disconnected trials across years are evaluated, i.e. year-wise with kinship approach (A1K), (iv) if only data from one selection cycle is available (TS_1_) there is a loss in *ρ*_*GP*_ with no options to improve via kinship or other models, (iv) predictive abilities improved in scenarios where TS and VS were more related (1P-scenario), and (v) pre-selection of top-yielding genotypes is recommended in cases where several single-year data are available within selection cycles and in such cases, the use of the kinship to model *GY* is also advisable.

## Additional file

Additional file 1 Supplementary Figures and Tables. (PDF 1066 kb)

## Abbreviations

BLUE: Best linear unbiased estimator
GCA: General combining ability
GEBV: Genomic estimated breeding value
GER: German dataset
GER&PL: German and Polish datasets pooled together
GP: Genomic prediction
GP-CV: Cross validation
GP-FV: Forward validation
*GY*: Genotype-by-year
LD: Linkage disequilibrium
MAF: Minor allele frequency
MET: Multi-environment trial
PCA: Principal component analysis
PL: Polish dataset
QTL: Quantitative trait loci
REML: Restricted maximum likelihood
RR-BLUP: Ridge regression-best linear unbiased predictor
SNP: Single nucleotide polymorphism
TS: Training set
VS: Validation set
*ρ*_*GP*_: Predictive ability
