# Supplementary material for: Genomic prediction in early selection stages using multi-year data in a hybrid rye breeding program
Source: BMC Genet. 2017 May 31;18:51. doi: 10.1186/s12863-017-0512-8 (PMC5452640; doi:10.1186/s12863-017-0512-8)
Supplement: Additional file 1 — Supplementary Figures and Tables. (PDF 1066 kb) [file 12863_2017_512_MOESM1_ESM.pdf]

Genomic prediction in early selection stages using multi-year data in a hybrid rye breeding program

Angela-Maria Bernal-Vasquez[1] Andres Gordillo[2] Malthe Schmidt[2]
Hans-Peter Piepho[1,3]

Additional file 1

Journal: BMC Genetics

[1]Biostatistics Unit, Institute of Crop Science, University of Hohenheim, Fruwirthstrasse 23, 70599 Stuttgart, Germany
[2]KWS-LOCHOW GMBH, Ferdinand-von-Lochow-Strasse 5, 29303, Bergen, Germany
[3]Corresponding author. Tel.: +49 711 459 22386; fax: +49 711 459 22297.
E-mail address: piepho@uni-hohenheim.de.

1 Complete selection breeding program

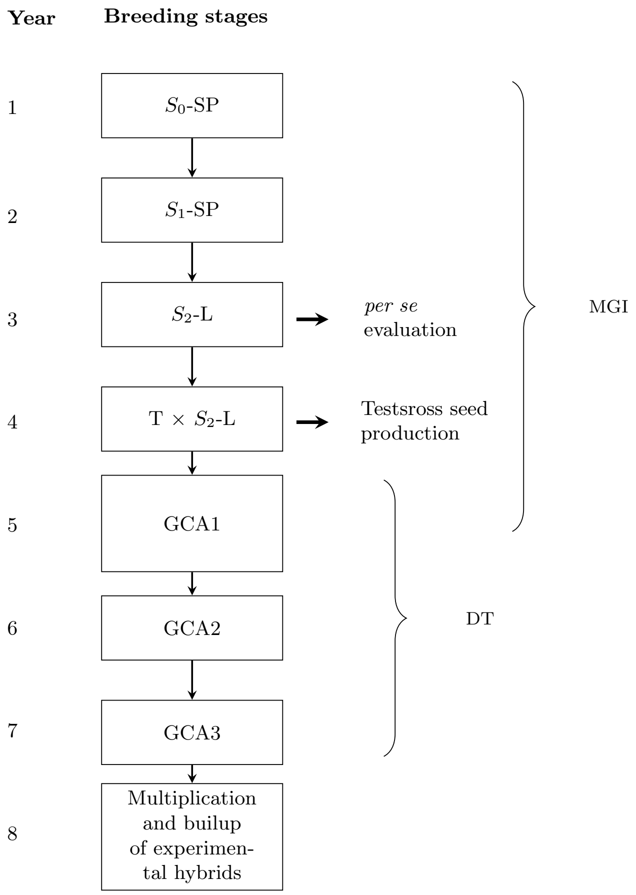

Figure S1: **Flow diagram of a complete selection cycle of the pollen parent pool.** S_x = selfing generation x, SP = single plant, L = line, T = tester, GCAX = general combining ability X trial, MGI = minimum generation interval, DT = datasets used

2 Diagrams of prediction scenarios

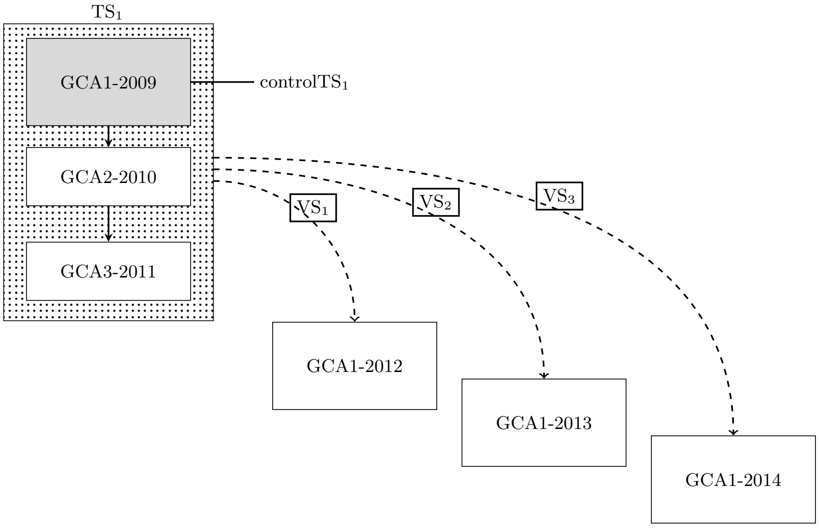

Figure S2: **Diagram of the first scenario.** TS_1 with dotted background and control set ($controlTS_1$) filled in gray. Arrows represent the prediction goals VS_1, VS_2 and VS_3.

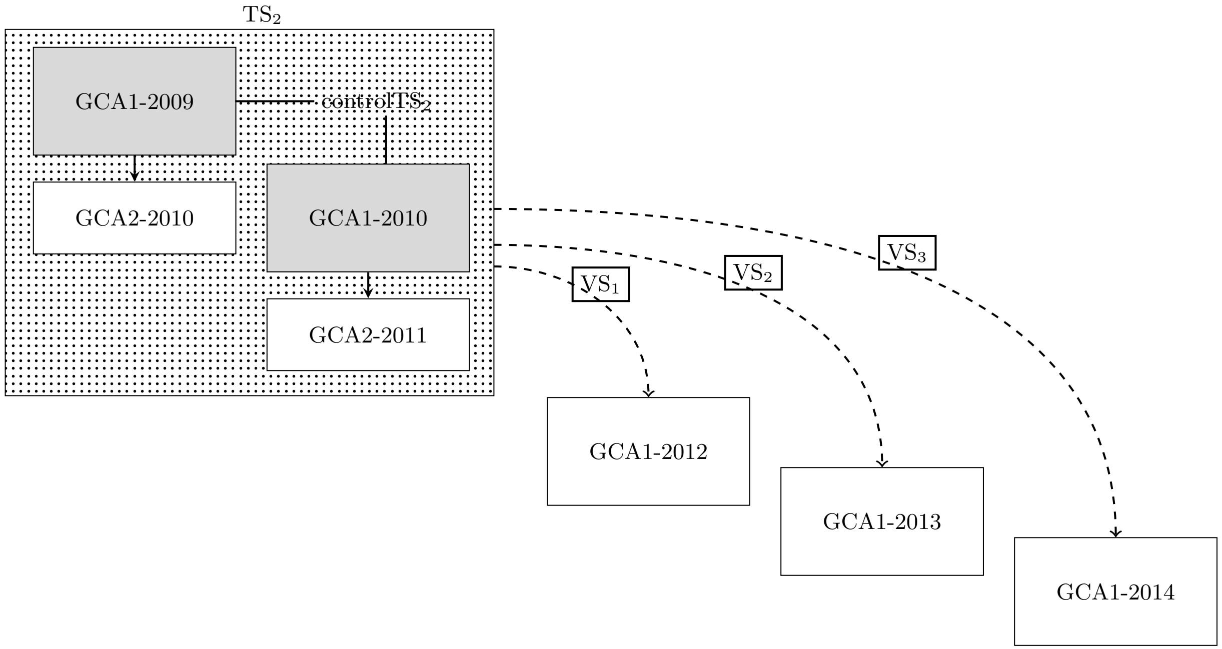

Figure S3: **Diagram of the second scenario.** TS$_2$ with dotted background and control set (controlTS$_2$) filled in gray. Arrows represent the prediction goals VS$_1$, VS$_2$ and VS$_3$.

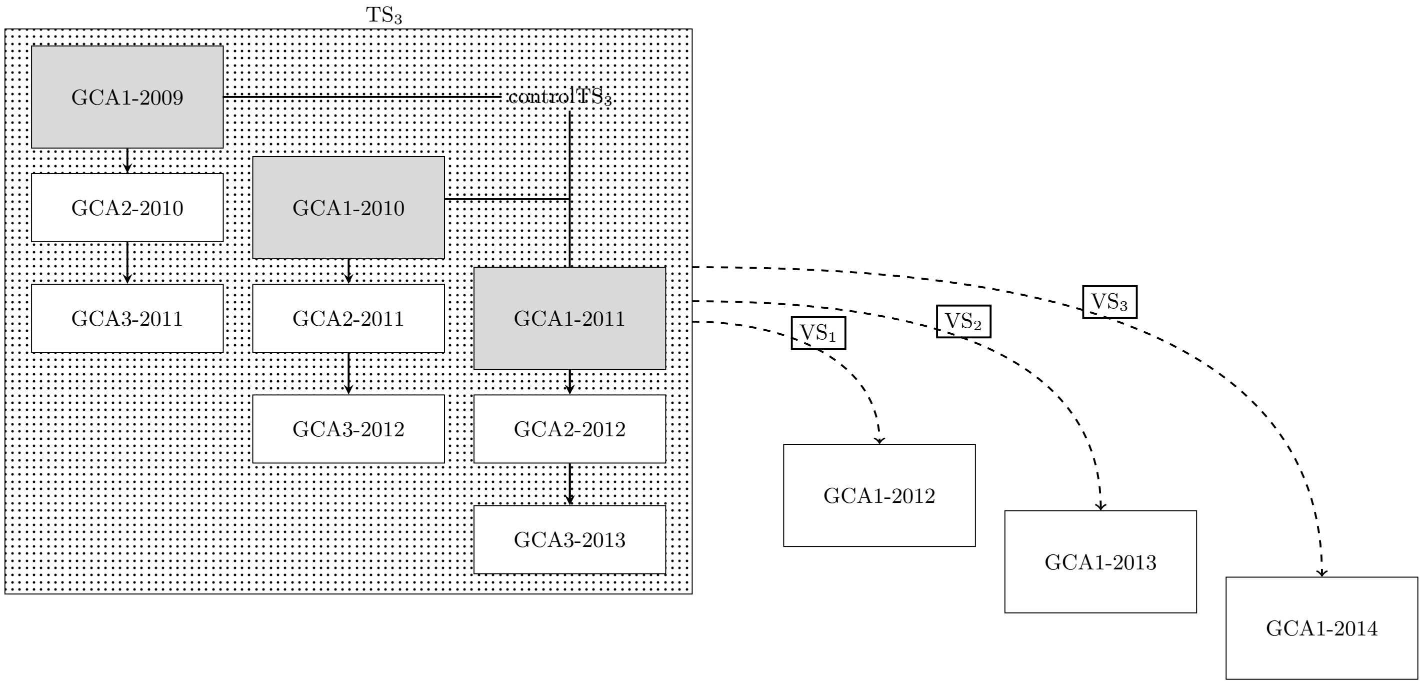

Figure S4: **Diagram of the third scenario.** TS_3 with dotted background and control set (controlTS_3) filled in gray. Arrows represent the prediction goals VS_1, VS_2 and VS_3.

3 Number of locations and Location-year combinations

Table S1: Number of locations (L) and year-location combinations (YL) for the German (GER) and the Polish (PL) datasets. Last column shows the ratio between YL and L

Program	TS	VS	L	YL	YL/L
GER	TS_1	VS_1	25	28	1.1
GER	TS_1	VS_2	24	31	1.3
GER	TS_1	VS_3	23	32	1.4
PL	TS_1	VS_1	23	23	1.0
PL	TS_1	VS_2	23	25	1.1
PL	TS_1	VS_3	22	26	1.2
GER	TS_2	VS_1	22	31	1.4
GER	TS_2	VS_2	21	29	1.4
GER	TS_2	VS_3	20	30	1.5
PL	TS_2	VS_1	21	25	1.2
PL	TS_2	VS_2	20	23	1.2
PL	TS_2	VS_3	19	24	1.3
GER	TS_3	VS_1	30	55	1.8
GER	TS_3	VS_2	29	56	1.9
GER	TS_3	VS_3	29	65	2.2
PL	TS_3	VS_1	29	49	1.7
PL	TS_3	VS_2	28	50	1.8
PL	TS_3	VS_3	28	59	2.1

TS_1: GCA1-2009 + GCA2-2010 + GCA3-2011, TS_2: GCA1-2009 + GCA2-2010 + GCA1-2010 + GCA2-2011, TS_3: GCA1-2009 + GCA2-2010 + GCA3-2011 + GCA1-2010 + GCA2-2011 + GCA3-2012 + GCA1-2011 + GCA2-2012 + GCA3-2013, VS_1: GCA1-2012, VS_2: GCA1-2013, VS_3: GCA1-2014.

4 Asymptotic correlation and variance-covariance matrices

Table S2: Asymptotic correlation (lower diagonal) and covariance (upper diagonal) matrix for TS_1-VS_3 German and Polish (GER&PL) dataset. TS_1: GCA1-2009 + GCA2-2010 + GCA3-2011, VS_3: GCA1-2014. The factors are genotypes (G), testers (T), years (Y) and locations (L).

	G	*GY*	*Y*	*L*	*GL*	*TL*	*GTL*	*GT*	*TY*	*GTY*	*YL*	*GYL*	*TYL*	*GTYL*
G	1	-0.4388	0.0102	0.0048	-0.0258	0	0.0016	-0.03	0	0.0279	-0.0008	0.0241	-0.0003	-0.0024
GY	**-0.8747**	1	-0.0101	-0.0069	0.0247	0	-0.002	0.027	0	-0.0314	0.0023	-0.0291	0.0005	0.0049
Y	0.4842	**-0.4283**	1	-103.93	0.0367	0	-0.0074	0.0442	0	-0.0447	-73.5891	-0.0388	0.0507	0.0079
L	0.0001	-0.0001	-0.0219	1	-0.0241	0	-0.0002	-0.0025	0	0.0025	-682.792	0.0226	-1.7145	0.0013
GL	-0.0553	0.0596	0.0009	-0.0005	1	0	-0.163	0.0094	0	-0.0093	-0.015	-0.3634	-0.0003	0.1556
TL	-	-	-	-	-	1	0	0	0	0	0	0	0	0
GTL	0.0022	-0.0032	-0.0001	0	**-0.2809**	-	1	-0.064	0	0.0629	0.0004	0.1544	0.0014	-0.8584
GT	-0.1106	0.112	0.0019	-0.0001	0.7852	-	**-0.1898**	1	0	-0.1247	-0.0056	-0.0095	0.0016	0.0629
TY	-	-	-	-	-	-	-	-	1	0	0	0	0	0
GTY	0.1022	-0.129	-0.0019	0.0001	-0.0413	-	0.1849	**-0.9491**	0	1	0.006	0.012	-0.0016	-0.0682
YL	-3E-05	0.0001	-0.0326	**-0.2556**	-0.0007	-	1.2E-05	-0.0004	0	0.0005	1	0.0135	-10.6168	-0.0001
GYL	0.0505	-0.0686	-0.001	0.0005	**-0.9229**	-	0.2601	-0.0414	0	0.0519	0.0006	1	0.0008	-0.1798
TYL	-0.0001	0.0002	0.0002	-0.0058	-0.0001	-	0.0004	0.0012	0	-0.0011	-0.0759	0.0003	1	-0.0021
GTYL	-0.0033	0.0078	0.0001	1.8E-05	0.2665	-	**-0.9758**	0.1851	0	**-0.1991**	0	**-0.3010**	-0.0006	1

Table S3: Asymptotic correlation (lower diagonal) and covariance (upper diagonal) matrix for TS_1-VS_2 German and Polish (GER&PL) dataset. TS_1: GCA1-2009 + GCA2-2010 + GCA3-2011, VS_2: GCA1-2013. The factors are genotypes (G), testers (T), years (Y) and locations (L).

	G	*GY*	*Y*	*L*	*GL*	*TL*	*GTL*	*GT*	*TY*	*GTY*	*YL*	*GYL*	*TYL*	*GTYL*
G	1	-0.2593	0.0102	-0.0003	-0.0131	0	0.0045	-0.0564	0	0.0509	0.0033	0.0125	-0.0008	-0.0043
GY	**-0.9082**	1	-0.0097	-0.0005	0.0131	0	-0.0042	0.0499	0	-0.0546	-0.0039	-0.0167	0.0009	0.0066
Y	0.3735	**-0.3420**	1	-200.189	0.0207	0	-0.0004	0.0607	0	-0.0639	-71.0256	-0.023	0.0358	0.0018
L	0.0000	0.0000	-0.0454	1	-0.0231	0	-0.0026	0.0027	0	-0.0021	-1206.04	0.0211	0.0789	0.0038
GL	-0.051	0.0531	0.0007	-0.0007	1	0	-0.1314	0.0081	0	-0.0084	-0.0095	-0.2118	-0.0003	0.1268
TL	-	-	-	-	-	1	0	0	0	0	0	0	0	0
GTL	0.0095	-0.0092	0.0000	0.0000	**-0.3215**	-	1	-0.0539	0	0.0529	0.0016	0.128	0.0012	-0.7415
GT	-0.238	0.2191	0.0021	0.0001	0.6708	-	-0.1431	1	0	-0.1772	-0.0112	-0.0095	0.0028	0.0536
TY	-	-	-	-	-	-	-	-	1	0	0	0	0	0
GTY	0.2171	-0.2420	-0.0022	-0.0001	-0.0415	-	0.1419	**-0.9470**	-	1	0.0112	0.0107	-0.0027	-0.0578
YL	0.0001	-0.0002	-0.0217	**-0.3748**	-0.0004	-	3.8E-05	-0.0005	-	0.0005	1	0.0094	-14.4578	-0.0015
GYL	0.0459	-0.0638	-0.0007	0.0006	**-0.8986**	-	0.2956	-0.0436	-	0.0497	0.0004	1	0.0008	-0.1544
TYL	-0.0003	0.0004	0.0001	0.0003	-0.0001	-	0.0003	0.0014	-	-0.0014	-0.0664	0.0003	1	-0.0019
GTYL	-0.009	0.0144	0.0000	0.0001	0.3059	-	**-0.9736**	0.1403	-	-0.1527	0.0000	-0.3515	-0.0005	1

Table S4: Asymptotic correlation (lower diagonal) and covariance (upper diagonal) matrix for TS_1-VS_1:GCA1-2012 German and Polish (GER&PL) dataset. TS_1: GCA1-2009 + GCA2-2010 + GCA3-2011, VS_1: GCA1-2012. The factors are genotypes (G), testers (T), years (Y) and locations (L).

	G	GY	Y	L	GL	TL	GTL	GT	TY	GTY	YL	GYL	TYL	$GTYL$
G	1	0	0	0	0	0	0	0	0	0	0	0	0	0
GY	-	1	-0.0042	0.0062	0	-0.0006	0.0035	-0.0008	0	-0.0074	-0.0003	-0.0076	0.0004	0
Y	-	-0.0002	1	-71.8508	0	11.8454	0.0008	0.1002	0	-0.0979	-40.1089	-0.0006	-11.9180	0
L	-	0.0003	-0.0224	1	0	4.5978	-0.0015	-0.0023	0	0.0026	-357.1928	-0.0009	-5.6630	0
GL	-	-	-	-	1	0	0	0	0	0	0	0	0	0
TL	-	-0.0001	0.0158	0.0055	-	1	-0.0028	-0.0194	0	0.0202	-23.6140	0.0018	-182.2474	0
GTL	-	0.0546	0.0001	-0.0001	-	-0.0010	1	-0.0027	0	-0.0044	0.0023	-0.0240	0.0005	0
GT	-	-0.0063	0.0049	-0.0001	-	-0.0036	-0.0371	1	0	-0.1423	-0.0021	0.0016	0.0207	0
TY	-	-	-	-	-	-	-	-	1	0	0	0	0	0
GTY	-	-0.0557	-0.0046	0.0001	-	0.0037	-0.0571	**-0.9440**	-	1	0.0018	0.0015	-0.0210	0
YL	-	0.0000	-0.0268	**-0.2138**	-	-0.0605	0.0004	-0.0002	-	0.0002	1	-0.0027	12.0806	0
GYL	-	-0.1057	-0.0001	-0.0001	-	0.0006	**-0.5756**	0.0196	-	0.0173	-0.0004	1	-0.0003	0
TYL	-	0.0001	-0.0163	-0.0069	-	**-0.9551**	0.0002	0.0040	-	-0.0039	0.0317	-0.0001	1	0
$GTYL$	-	-	-	-	-	-	-	-	-	-	-	-	-	1

Table S5: Asymptotic correlation (lower diagonal) and covariance (upper diagonal) matrix for TS_2-VS_3:GCA1-2014 German and Polish (GER&PL) dataset. TS_2: GCA1-2009 + GCA2-2010 + GCA1-2010 + GCA2-2011, VS_3: GCA1-2014. The factors are genotypes (G), testers (T), years (Y) and locations (L).

	G	GY	Y	L	GL	TL	GTL	GT	TY	GTY	YL	GYL	TYL	$GTYL$
G	1	-0.1791	0.0089	-0.0124	-0.0373	0	0.0115	-0.0365	0	0.0336	0.0102	0.0373	-0.0081	-0.0125
GY	**-0.8111**	1	-0.0090	0.0086	0.0373	0	-0.0119	0.0338	0	-0.0369	-0.0069	-0.0420	0.0069	0.0154
Y	0.2371	**-0.1994**	1	12.2686	0.0398	0	-0.0149	0.0045	0	-0.0064	-74.2372	-0.0397	-0.3763	0.0140
L	-0.0003	0.0003	0.0032	1	-0.0291	0	-0.0058	0.0031	0	-0.0025	-458.0833	0.0284	-0.3409	0.0062
GL	-0.0915	0.1094	0.0010	-0.0005	1	0	-0.3417	0.0147	0	-0.0142	0.0090	-0.6169	-0.0015	0.3356
TL	-	-	-	-	-	1	0	0	0	0	0	0	0	0
GTL	0.0275	-0.0342	-0.0004	-0.0001	**-0.5319**	-	1	-0.0385	0	0.0378	-0.0097	0.3364	0.0023	-0.6483
GT	**-0.2562**	0.2843	0.0003	0.0002	1.1782	-	-0.1716	1	0	-0.0710	-0.0042	-0.0143	0.0001	0.0373
TY	-	-	-	-	-	-	-	-	1	0	0	0	0	0
GTY	0.2345	**-0.3084**	-0.0004	-0.0001	-0.0644	-	0.1676	**-0.9205**	-	1	0.0044	0.0170	-0.0002	-0.0433
YL	0.0006	-0.0005	-0.0471	**-0.2043**	0.0004	-	-3.9E-04	-0.0005	-	0.0005	1	-0.0095	-3.1882	0.0095
GYL	0.0900	-0.1214	-0.0010	0.0005	**-0.9657**	-	0.5157	-0.0641	-	0.0758	-0.0004	1	0.0013	-0.3594
TYL	-0.0075	0.0076	-0.0035	-0.0022	-0.0009	-	0.0013	0.0002	-	-0.0003	-0.0496	0.0008	1	-0.0021
$GTYL$	-0.0294	0.0436	0.0003	0.0001	0.5128	-	**-0.9701**	0.1633	-	**-0.1884**	0.0004	**-0.5409**	-0.0012	1

Table S6: Asymptotic correlation (lower diagonal) and covariance (upper diagonal) matrix for TS_2-VS_2:GCA1-2013 German and Polish (GER&PL) dataset. TS_2: GCA1-2009 + GCA2-2010 + GCA1-2010 + GCA2-2011, VS_2: GCA1-2013. The factors are genotypes (G), testers (T), years (Y) and locations (L).

	Y	G	GY	L	GL	TL	GTL	GT	TY	GTY	YL	GYL	TYL	$GTYL$
G	1	-0.1341	0.0084	-0.0123	-0.0243	0	0.0106	-0.0475	0	0.0425	0.0110	0.0240	-0.0060	-0.0106
GY	**-0.8469**	1	-0.0080	0.0097	0.0245	0	-0.0107	0.0429	0	-0.0460	-0.0093	-0.0281	0.0052	0.0137
Y	0.1754	-0.1511	1	3.5213	0.0199	0	-0.0064	0.0044	0	-0.0078	-120.6435	-0.0203	-0.3188	0.0063
L	-0.0005	0.0004	0.0011	1	-0.0195	0	-0.0107	0.0045	0	-0.0036	-837.9200	0.0187	0.0361	0.0112
GL	-0.0913	0.1020	0.0006	-0.0005	1	0	-0.2598	0.0117	0	-0.0115	0.0059	-0.3946	-0.0008	0.2552
TL	-	-	-	-	-	1	0	0	0	0	0	0	0	0
GTL	0.0345	-0.0388	-0.0002	-0.0002	-0.5584	-	1	-0.0325	0	0.0320	-0.0063	0.2563	0.0018	-0.5317
GT	**-0.3540**	0.3555	0.0003	0.0002	0.9334	-	-0.1385	1	0	-0.0933	-0.0064	-0.0117	0.0004	0.0316
TY	-	-	-	-	-	-	-	-	1	0	0	0	0	0
GTY	0.3204	**-0.3847**	-0.0005	-0.0002	-0.0570	-	0.1378	**-0.9229**	-	1	0.0064	0.0137	-0.0004	-0.0367
YL	0.0006	-0.0006	-0.0572	**-0.3189**	0.0002	-	-2.0E-04	-0.0005	-	0.0005	1	-0.0063	-4.4431	0.0063
GYL	0.0878	-0.1147	-0.0006	0.0005	**-0.9563**	-	0.5378	-0.0564	-	0.0666	-0.0002	1	0.0007	-0.2779
TYL	-0.0058	0.0057	-0.0026	0.0002	-0.0005	-	0.0010	0.0004	-	-0.0005	-0.0430	0.0004	1	-0.0016
$GTYL$	-0.0337	0.0484	0.0002	0.0002	0.5361	-	**-0.9671**	0.1319	-	-0.1547	0.0002	-0.5700	-0.0009	1

Table S7: Asymptotic correlation (lower diagonal) and covariance (upper diagonal) matrix for TS_2-VS_1:GCA1-2012 German and Polish (GER&PL) dataset. TS_2: GCA1-2009 + GCA2-2010 + GCA1-2010 + GCA2-2011, VS_1: GCA1-2012. The factors are genotypes (G), testers (T), years (Y) and locations (L).

	G	GY	Y	L	GL	TL	GTL	GT	TY	GTY	YL	GYL	TYL	$GTYL$
G	1	-0.1526	0.2375	0.0016	-0.0222	0	0.0086	-0.0326	0	0.0294	0.0011	0.0210	-0.0067	-0.0083
GY	**-0.7924**	1	-0.1947	0.0024	0.0222	0	-0.0089	0.0296	0	-0.0330	-0.0013	-0.0258	0.0053	0.0116
Y	0.0089	-0.0087	1	0.7995	0.0208	0	-0.0048	0.0057	0	-0.0084	-46.5272	-0.0219	-0.3627	0.0045
L	0.0001	0.0001	0.0003	1	-0.0164	0	-0.0050	0.0007	0	-0.0003	-243.4461	0.0143	-0.2184	0.0051
GL	-0.0764	0.0908	0.0006	-0.0005	1	0	-0.2234	0.0107	0	-0.0103	0.0042	-0.3623	-0.0006	0.2200
TL	-	-	-	-	-	1	0	0	0	0	0	0	0	0
GTL	0.0259	-0.0319	-0.0001	-0.0001	**-0.5329**	-	1	-0.0288	0	0.0282	-0.0069	0.2200	0.0016	-0.4705
GT	-0.2527	0.2739	0.0004	0.0000	0.0652	-	-0.1548	1	0	-0.0667	-0.0021	-0.0099	0.0002	0.0276
TY	-	-	-	-	-	-	-	-	1	0	0	0	0	0
GTY	0.2259	**-0.3026**	-0.0006	0.0000	-0.0624	-	0.1507	**-0.9122**	-	1	0.0020	0.0124	-0.0001	-0.0329
YL	0.0001	-0.0001	-0.0343	-0.1802	0.0003	-	-0.0004	-0.0003	-	0.0003	1	-0.0043	-3.3215	0.0071
GYL	0.0703	-0.1033	-0.0006	0.0004	**-0.9589**	-	0.5123	-0.0593	-	0.0737	-0.0003	1	0.0005	-0.2364
TYL	-0.0063	0.0060	-0.0029	-0.0018	-0.0004	-	0.0011	0.0003	-	-0.0002	-0.0615	0.0004	1	-0.0015
$GTYL$	-0.0247	0.0409	0.0001	0.0001	0.5149	-	**-0.9685**	0.1455	-	-0.1719	0.0004	**-0.5399**	-0.0009	1

Table S8: Asymptotic correlation (lower diagonal) and covariance (upper diagonal) matrix for TS_3-VS_3:GCA1-2014 German and Polish (GER&PL) dataset. TS_3: GCA1-2009 + GCA2-2010 + GCA3-2011 + GCA1-2010 + GCA2-2011 + GCA3-2012 + GCA1-2011 + GCA2-2012 + GCA3-2013, VS_3: GCA1-2014. The factors are genotypes (G), testers (T), years (Y) and locations (L).

	G	GY	Y	L	GL	TL	GTL	GT	TY	GTY	YL	GYL	TYL	$GTYL$
G	1	-0.0427	-0.0394	-0.0046	-0.0045	0.0017	-0.0004	-0.0108	0	0.0091	0.0021	0.0039	-0.0011	0.0001
GY	**-0.6425**	1	0.0300	0.0028	0.0038	-0.0009	-0.0001	0.0089	0	-0.0108	-0.0012	-0.0058	0.0006	0.0015
Y	-0.0027	0.0030	1	-11.3578	0.0015	-3.6709	0.0090	-0.0039	0	0.0029	-13.0843	-0.0009	2.9638	-0.0091
L	-0.0003	0.0002	-0.0042	1	-0.0005	-2.4570	-0.0026	0.0003	0	0.0000	-95.7799	0.0015	1.7234	0.0019
GL	-0.0544	0.0687	0.0001	0.0000	1	0.0005	-0.0342	0.0018	0	-0.0019	0.0042	-0.0649	-0.0004	0.0333
TL	0.0010	-0.0008	-0.0150	-0.0082	0.0003	1	0.0006	-0.0010	0	0.0000	1.3346	0.0015	-22.8760	-0.0018
GTL	-0.0028	-0.0013	0.0004	-0.0001	**-0.2839**	0.0003	1	-0.0130	0	0.0129	-0.0020	0.0329	0.0001	-0.2099
GT	-0.1873	0.2286	-0.0005	0.0000	0.0371	-0.0010	-0.1531	1	0	-0.0297	-0.0007	-0.0018	0.0009	0.0126
TY	-	-	-	-	-	-	-	-	1	0	0	0	0	0
GTY	0.1601	**-0.2815**	0.0003	0.0000	-0.0398	0.0000	0.1538	**-0.8880**	-	1	0.0007	0.0029	0.0004	-0.0152
YL	0.0004	-0.0003	-0.0165	-0.0981	0.0009	0.0150	-0.0003	-0.0002	-	0.0002	1	-0.0044	-4.4463	0.0020
GYL	0.0430	-0.0940	-0.0001	0.0001	**-0.8576**	0.0010	0.2468	-0.0333	-	0.0553	-0.0009	1	-0.0016	-0.0445
TYL	-0.0007	0.0006	0.0127	0.0060	-0.0003	**-0.8751**	0.0000	0.0010	-	0.0004	-0.0525	-0.0011	1	0.0007
$GTYL$	0.0006	0.0149	-0.0004	0.0001	0.2688	-0.0007	**-0.9604**	0.1435	-	-0.1768	0.0003	-0.3239	0.0003	1

Table S9: Asymptotic correlation (lower diagonal) and covariance (upper diagonal) matrix for TS_3-VS_2:GCA1-2013 German and Polish (GER&PL) dataset. TS_3: GCA1-2009 + GCA2-2010 + GCA3-2011 + GCA1-2010 + GCA2-2011 + GCA3-2012 + GCA1-2011 + GCA2-2012 + GCA3-2013, VS_2: GCA1-2013. The factors are genotypes (G), testers (T), years (Y) and locations (L).

	G	GY	Y	L	GL	TL	GTL	GT	TY	GTY	YL	GYL	TYL	$GTYL$
G	1	-0.0489	-0.0526	0.0053	-0.0033	0.0038	-0.0001	-0.0116	0	0.0093	0.0018	0.0027	-0.0001	0.0001
GY	**-0.6967**	1	0.0438	-0.0054	0.0028	-0.0028	-0.0003	0.0087	0	-0.0110	-0.0020	-0.0047	-0.0003	0.0016
Y	-0.0037	0.0040	1	-10.9361	0.0002	-4.7179	0.0079	-0.0066	0	0.0051	-17.3793	-0.0001	3.8107	-0.0080
L	0.0003	-0.0004	-0.0042	1	-0.0001	-3.3662	-0.0035	-0.0011	0	0.0012	-131.2160	0.0006	2.6769	0.0034
GL	-0.0506	0.0550	0.0000	0.0000	1	0.0016	-0.0254	0.0013	0	-0.0015	0.0011	-0.0434	-0.0024	0.0248
TL	0.0023	-0.0021	-0.0176	-0.0110	0.0013	1	-0.0001	-0.0019	0	0.0005	2.5194	0.0002	-25.7527	-0.0008
GTL	-0.0010	-0.0031	0.0004	-0.0001	**-0.2712**	0.0000	1	-0.0118	0	0.0116	-0.0002	0.0246	0.0012	-0.1867
GT	**-0.1988**	0.1937	-0.0007	-0.0001	0.0316	-0.0017	-0.1408	1	0	-0.0311	-0.0016	-0.0014	0.0012	0.0113
TY	-	-	-	-	-	-	-	-	1	0	0	0	0	0
GTY	0.1659	**-0.2552**	0.0006	0.0001	-0.0366	0.0005	0.1447	**-0.8708**	-	1	0.0016	0.0025	0.0003	-0.0140
YL	0.0003	-0.0004	-0.0187	-0.1234	0.0003	0.0230	0.0000	-0.0004	-	0.0004	1	0.0040	-5.4092	-0.0036
GYL	0.0365	-0.0819	0.0000	0.0000	**-0.8136**	0.0002	0.2281	-0.0290	-	0.0536	0.0008	1	-0.0001	-0.0359
TYL	-0.0001	-0.0002	0.0152	0.0094	-0.0021	**-0.8720**	0.0005	0.0012	-	0.0004	-0.0528	-0.0001	1	-0.0001
$GTYL$	0.0011	0.0155	-0.0004	0.0001	0.2561	-0.0003	**-0.9560**	0.1306	-	-0.1677	-0.0004	**-0.3222**	0.0000	1

Table S10: Asymptotic correlation (lower diagonal) and covariance (upper diagonal) matrix for TS_3-VS_1:GCA1-2012 German and Polish (GER&PL) dataset. TS_3: GCA1-2009 + GCA2-2010 + GCA3-2011 + GCA1-2010 + GCA2-2011 + GCA3-2012 + GCA1-2011 + GCA2-2012 + GCA3-2013, VS_1: GCA1-2012. The factors are genotypes (G), testers (T), years (Y) and locations (L).

	G	GY	Y	L	GL	TL	GTL	GT	TY	GTY	YL	GYL	TYL	$GTYL$
G	1	-0.2622	-0.4926	0.0179	-0.0056	-0.0058	-0.0007	-0.0140	0.0002	0.0122	-0.0096	0.0052	0.0050	0.0005
GY	**-0.8915**	1	0.4666	-0.0146	0.0049	0.0054	0.0007	0.0118	-0.0007	-0.0148	0.0088	-0.0082	-0.0050	0.0012
Y	-0.0158	0.0153	1	-8.4482	-0.0009	-7.1051	0.0157	-0.0194	-2.3148	0.0149	-15.8806	-0.0016	6.4270	-0.0130
L	0.0006	-0.0005	-0.0027	1	-0.0126	-2.8175	0.0006	0.0003	-0.2087	0.0002	-118.7310	0.0130	2.1704	-0.0017
GL	-0.0351	0.0310	-0.0001	-0.0008	1	-0.0009	-0.0455	0.0023	-0.0009	-0.0025	0.0114	-0.0821	-0.0012	0.0445
TL	-0.0022	0.0020	-0.0255	-0.0106	-0.0006	1	-0.0006	-0.0001	1.8393	0.0005	1.8097	0.0032	-19.5968	-0.0018
GTL	-0.0027	0.0028	0.0006	0.0000	-0.3169	-0.0002	1	-0.0160	-0.0007	0.0159	-0.0048	0.0437	0.0027	-0.2373
GT	-0.1163	0.1001	-0.0016	0.0000	0.0365	-0.0001	-0.1481	1	-0.0017	-0.0431	-0.0015	-0.0021	0.0001	0.0151
TY	0.0003	-0.0009	-0.0275	-0.1586	-0.0020	0.2532	-0.0010	-0.0051	1	0.0029	0.1432	0.0006	-2.0138	0.0002
GTY	0.1014	-0.1257	0.0012	0.0000	-0.0392	0.0004	0.1472	**-0.8951**	0.0088	1	0.0011	0.0038	0.0000	-0.0190
YL	-0.0010	0.0009	-0.0153	-0.1190	0.0021	0.0201	-0.0005	-0.0004	0.0053	0.0003	1	-0.0106	-4.2475	0.0045
GYL	0.0296	-0.0473	-0.0001	0.0007	**-0.8693**	0.0020	0.2753	-0.0293	0.0012	0.0542	-0.0018	1	-0.0020	-0.0568
TYL	0.0020	-0.0020	0.0244	0.0086	-0.0009	**-0.8614**	0.0012	0.0001	**-0.2934**	0.0000	-0.0500	-0.0013	1	-0.0006
$GTYL$	0.0019	0.0043	-0.0005	-0.0001	0.3016	-0.0007	**-0.9553**	0.1362	0.0003	-0.1720	0.0005	**-0.3480**	-0.0002	1

5 Predictive abilities of sampling scenarios

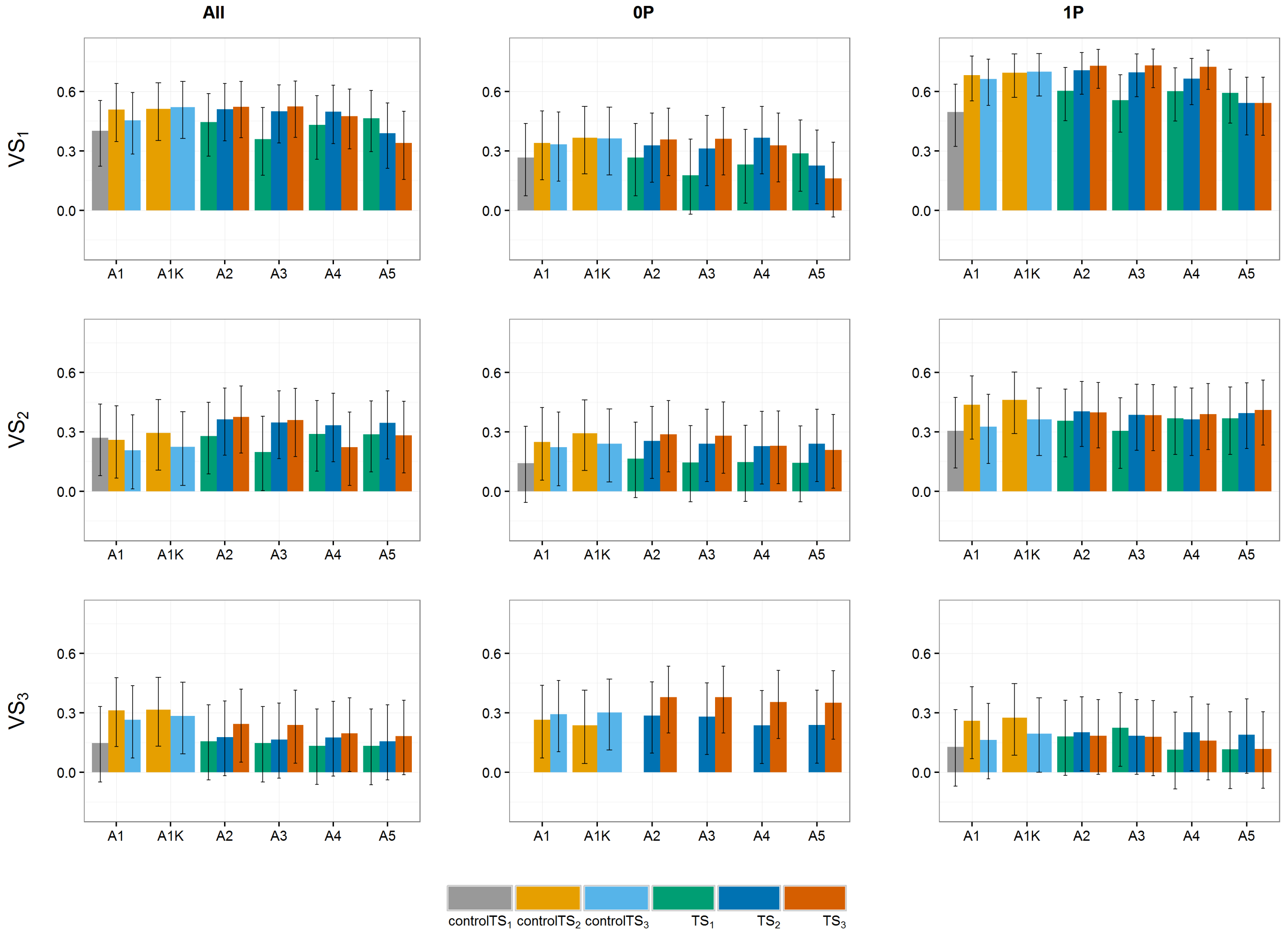

Figure S5: **Predictive abilities (y-axis) of the German dataset using VS-size of 100 genotypes for the three scenarios.** TS_1 and controlTS_1, TS_2 and controlTS_2, and TS_3 and controlTS_3 to predict the validation sets VS_1, VS_2 and VS_3 with All-, 0P- and 1P-scenarios. Black lines for each bar represent the 95% confidence intervals of the mean predictive ability. Year-wise approach (A1) and year-wise with kinship approach (A1K) were fitted to the control sets, approaches 2-stg-Kin (A2), 2-stg-Kin-het (A3), 3-stg-NoKin (A4) and 3-stg-Kin (A5) to the complete sets. TS_1: GCA1-2009 + GCA2-2010 + GCA3-2011, controlTS_1: GCA1-2009, TS_2: GCA1-2009 + GCA2-2010 + GCA1-2010 + GCA2-2011, controlTS_2: GCA1-2009 + GCA1-2010, TS_3: GCA1-2009 + GCA2-2010 + GCA3-2011 + GCA1-2010 + GCA2-2011 + GCA3-2012 + GCA1-2011 + GCA2-2012 + GCA3-2013, controlTS_3: GCA1-2009 + GCA1-2010 + GCA1-2011, VS_1: GCA1-2012, VS_2: GCA1-2013, VS_3: GCA1-2014.

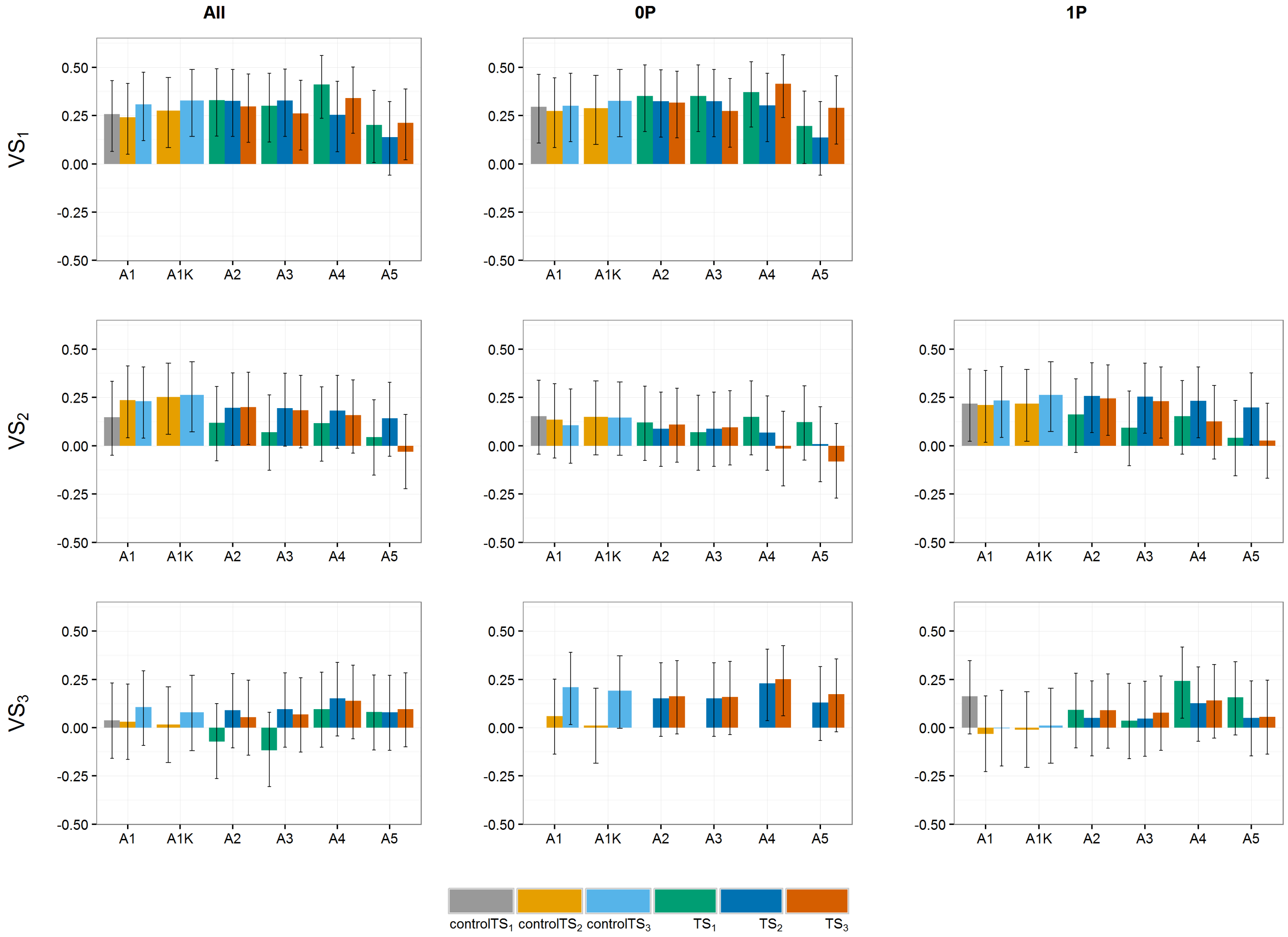

Figure S6: **Predictive abilities (y-axis) of the Polish dataset using VS-size of 100 genotypes for the three scenarios.** TS_1 and controlTS_1, TS_2 and controlTS_2, and TS_3 and controlTS_3 to predict the validation sets VS_1, VS_2 and VS_3 with All-, 0P- and 1P-scenarios. Black lines for each bar represent the 95% confidence intervals of the mean predictive ability. Year-wise approach (A1) and year-wise with kinship approach (A1K) were fitted to the control sets, approaches 2-stg-Kin (A2), 2-stg-Kin-het (A3), 3-stg-NoKin (A4) and 3-stg-Kin (A5) to the complete sets. TS_1: GCA1-2009 + GCA2-2010 + GCA3-2011, controlTS_1: GCA1-2009, TS_2: GCA1-2009 + GCA2-2010 + GCA1-2010 + GCA2-2011, controlTS_2: GCA1-2009 + GCA1-2010, TS_3: GCA1-2009 + GCA2-2010 + GCA3-2011 + GCA1-2010 + GCA2-2011 + GCA3-2012 + GCA1-2011 + GCA2-2012 + GCA3-2013, controlTS_3: GCA1-2009 + GCA1-2010 + GCA1-2011, VS_1: GCA1-2012, VS_2: GCA1-2013, VS_3: GCA1-2014.

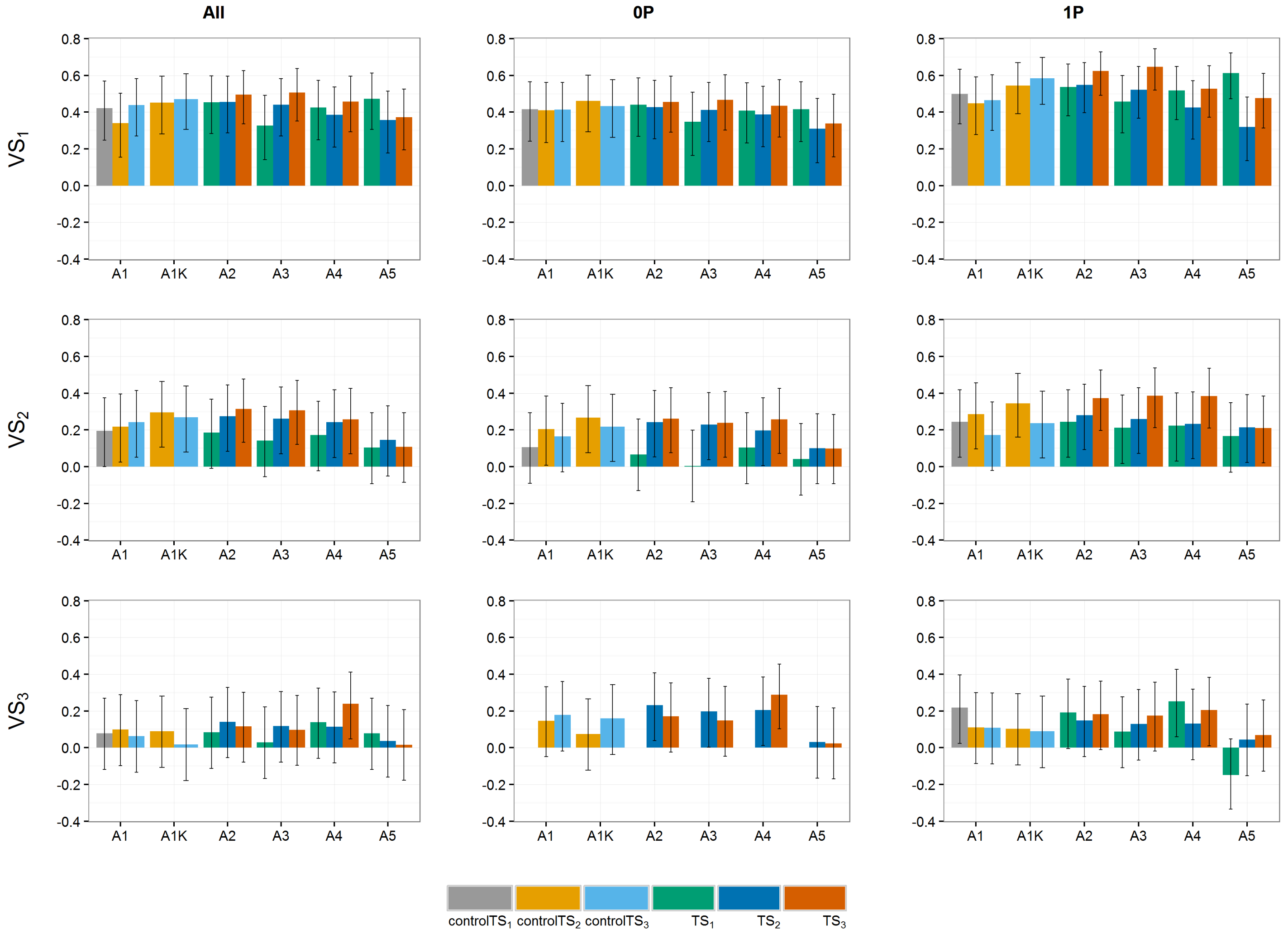

Figure S7: **Predictive abilities (y-axis) of the German and Polish dataset using VS-size of 100 genotypes for the three scenarios.** TS_1 and $controlTS_1$, TS_2 and $controlTS_2$, and TS_3 and $controlTS_3$ to predict the validation sets VS_1, VS_2 and VS_3 with All-, 0P- and 1P-scenarios. Black lines for each bar represent the 95% confidence intervals of the mean predictive ability. Year-wise approach (A1) and year-wise with kinship approach (A1K) were fitted to the control sets, approaches 2-stg-Kin (A2), 2-stg-Kin-het (A3), 3-stg-NoKin (A4) and 3-stg-Kin (A5) to the complete sets. TS_1: GCA1-2009 + GCA2-2010 + GCA3-2011, $controlTS_1$: GCA1-2009, TS_2: GCA1-2009 + GCA2-2010 + GCA1-2010 + GCA2-2011, $controlTS_2$: GCA1-2009 + GCA1-2010, TS_3: GCA1-2009 + GCA2-2010 + GCA3-2011 + GCA1-2010 + GCA2-2011 + GCA3-2012 + GCA1-2011 + GCA2-2012 + GCA3-2013, $controlTS_3$: GCA1-2009 + GCA1-2010 + GCA1-2011, VS_1: GCA1-2012, VS_2: GCA1-2013, VS_3: GCA1-2014.

6 PCA plots

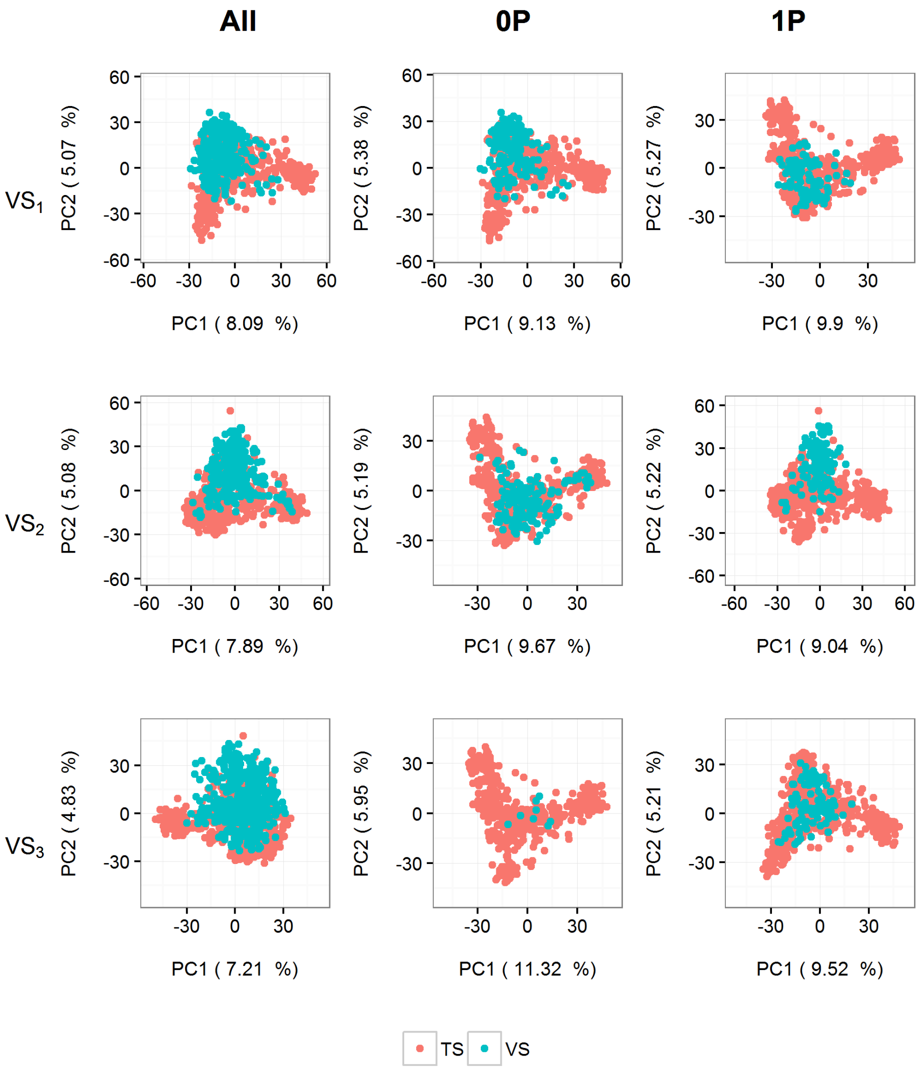

Figure S8: **Principal component (PC) plots for the German dataset between TS_1 and all VS.** TS_1 and relatedness scenarios (All-, 0P- and 1P-scenarios) for VS_1, VS_2 and VS_3. TS_1:GCA1-2009 + GCA2-2010 + GCA3-2011, VS_1:GCA1-2012, VS_2:GCA1-2013, VS_3:GCA1-2014.

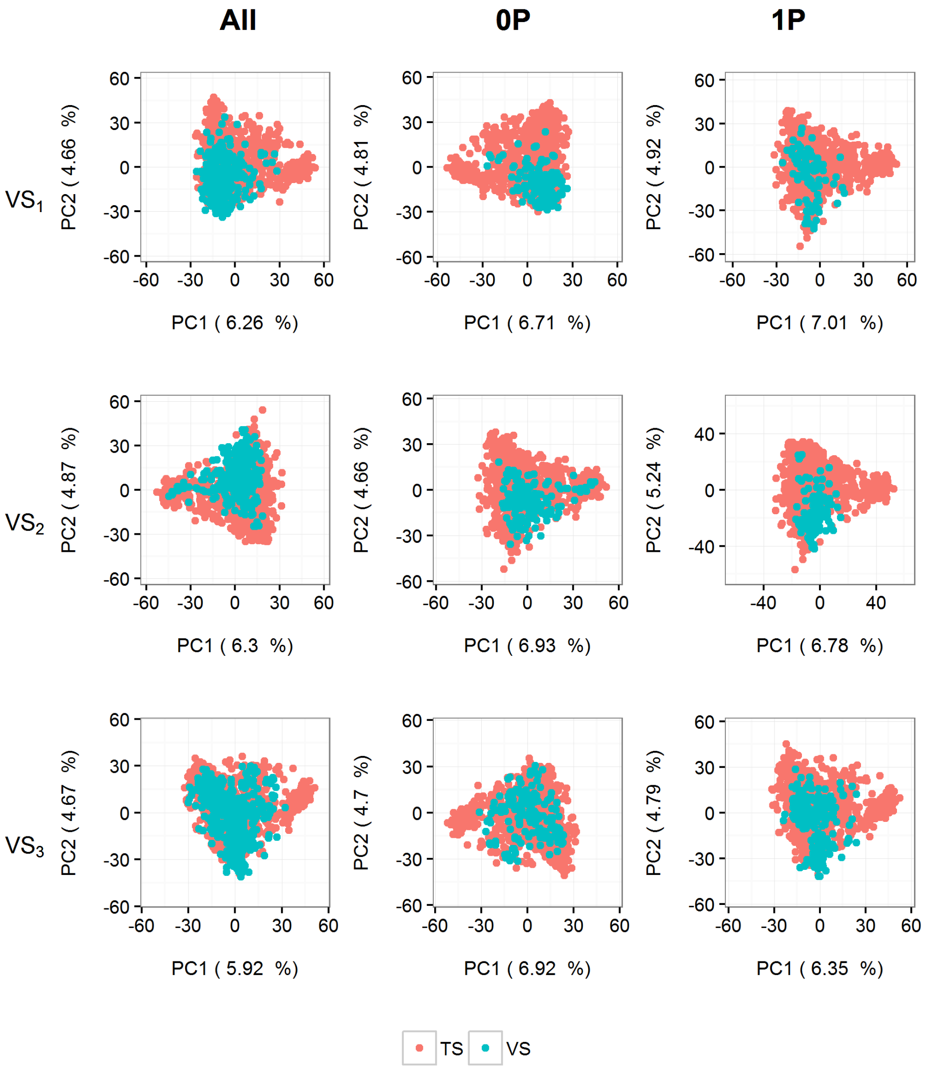

Figure S9: **Principal component (PC) plots for the German dataset between TS_2 and all VS.** TS_2 and relatedness scenarios (All-, 0P- and 1P-scenarios) for VS_1, VS_2 and VS_3. TS_2:GCA1-2009 + GCA2-2010 + GCA1-2010 + GCA2-2011, VS_1:GCA1-2012, VS_2:GCA1-2013, VS_3:GCA1-2014.

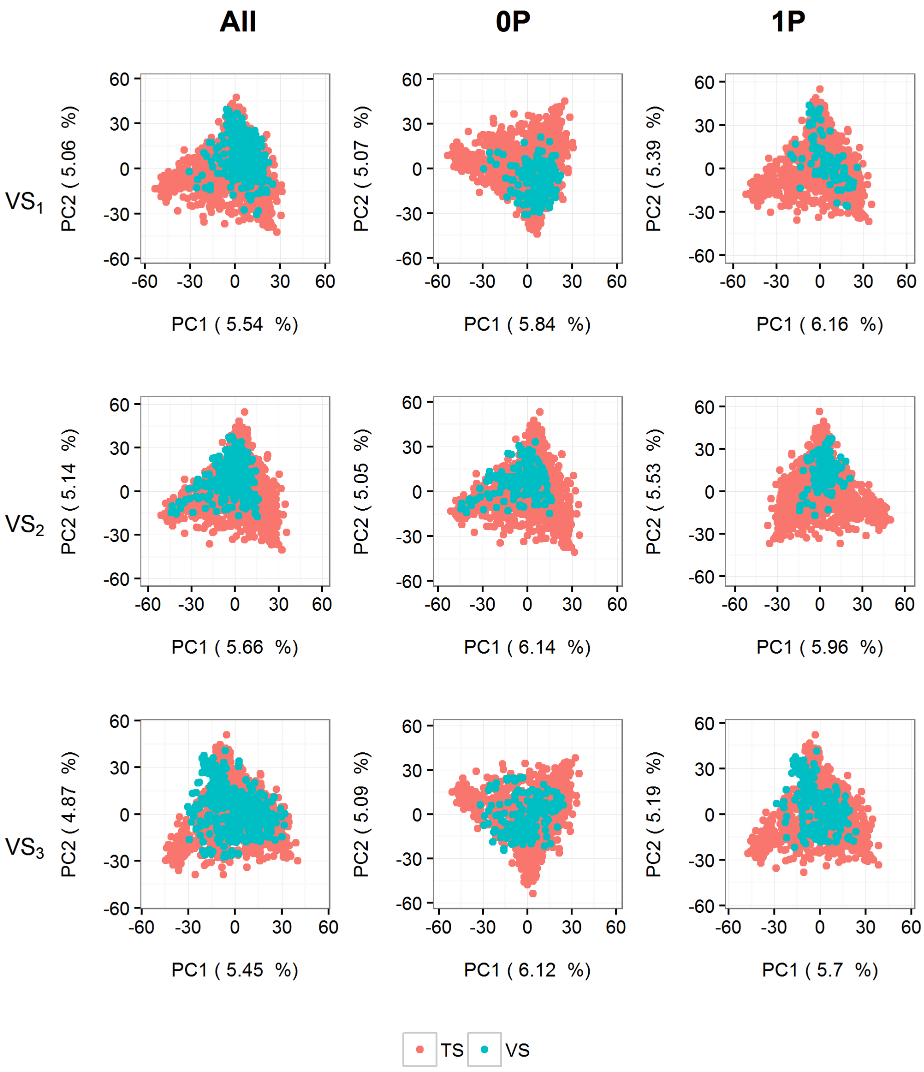

Figure S10: **Principal component (PC) plots for the German dataset between TS_3 and all VS.** TS_3 and relatedness scenarios (All-, 0P- and 1P-scenarios) for VS_1, VS_2 and VS_3. TS_3:GCA1-2009 + GCA2-2010 + GCA3-2011 + GCA1-2010 + GCA2-2011 + GCA3-2012 + GCA1-2011 + GCA2-2012 + GCA3-2013, VS_1:GCA1-2012, VS_2:GCA1-2013, VS_3:GCA1-2014.

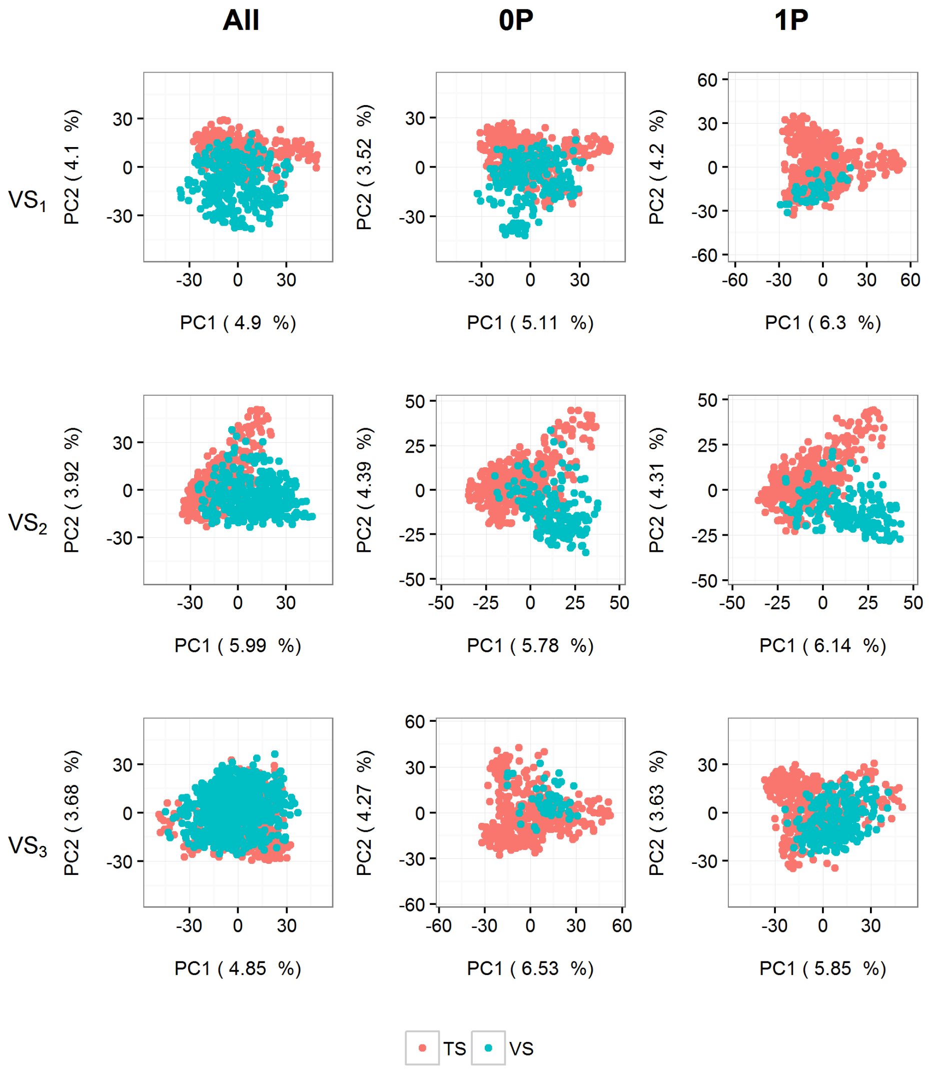

Figure S11: **Principal component (PC) plots for the Polish dataset between TS_1 and all VS.** TS_1 and relatedness scenarios (All-, 0P- and 1P-scenarios) for VS_1, VS_2 and VS_3. TS_1:GCA1-2009 + GCA2-2010 + GCA3-2011, VS_1:GCA1-2012, VS_2:GCA1-2013, VS_3:GCA1-2014.

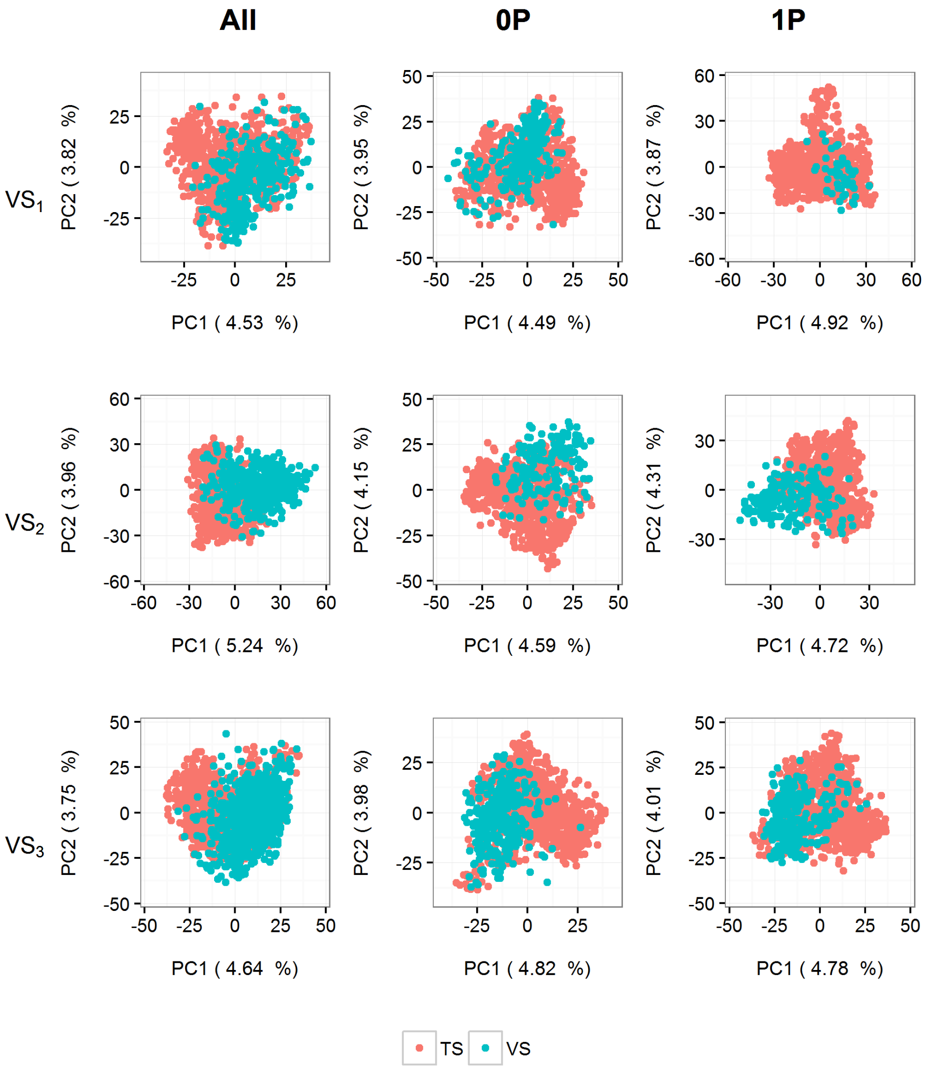

Figure S12: **Principal component (PC) plots for the Polish dataset between TS$_2$ and all VS.** TS$_2$ and relatedness scenarios (All-, 0P- and 1P-scenarios) for VS$_1$, VS$_2$ and VS$_3$. TS$_2$:GCA1-2009 + GCA2-2010 + GCA1-2010 + GCA2-2011, VS$_1$:GCA1-2012, VS$_2$:GCA1-2013, VS$_3$:GCA1-2014.

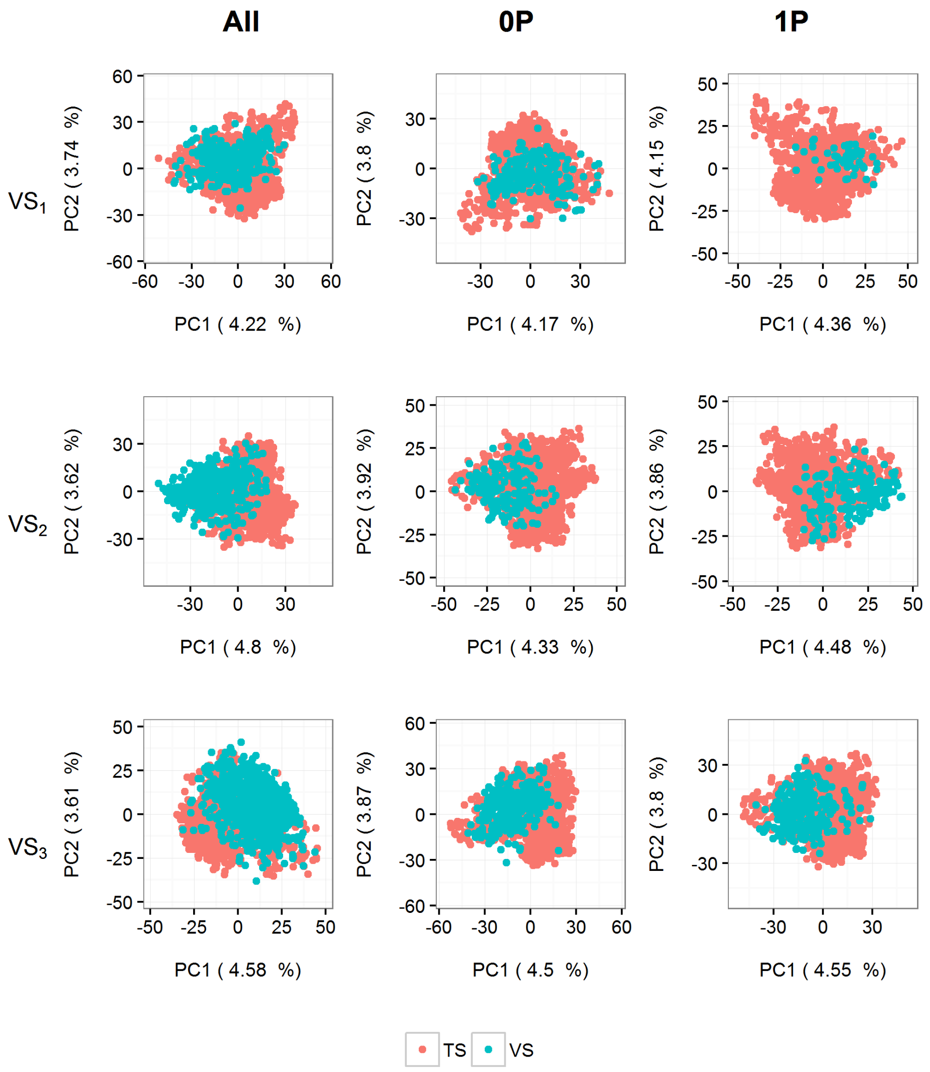

Figure S13: **Principal component (PC) plots for the Polish dataset between TS_3 and all VS.** TS_3 and relatedness scenarios (All-, 0P- and 1P-scenarios) for VS_1, VS_2 and VS_3. TS_3:GCA1-2009 + GCA2-2010 + GCA3-2011 + GCA1-2010 + GCA2-2011 + GCA3-2012 + GCA1-2011 + GCA2-2012 + GCA3-2013, VS_1:GCA1-2012, VS_2:GCA1-2013, VS_3:GCA1-2014.

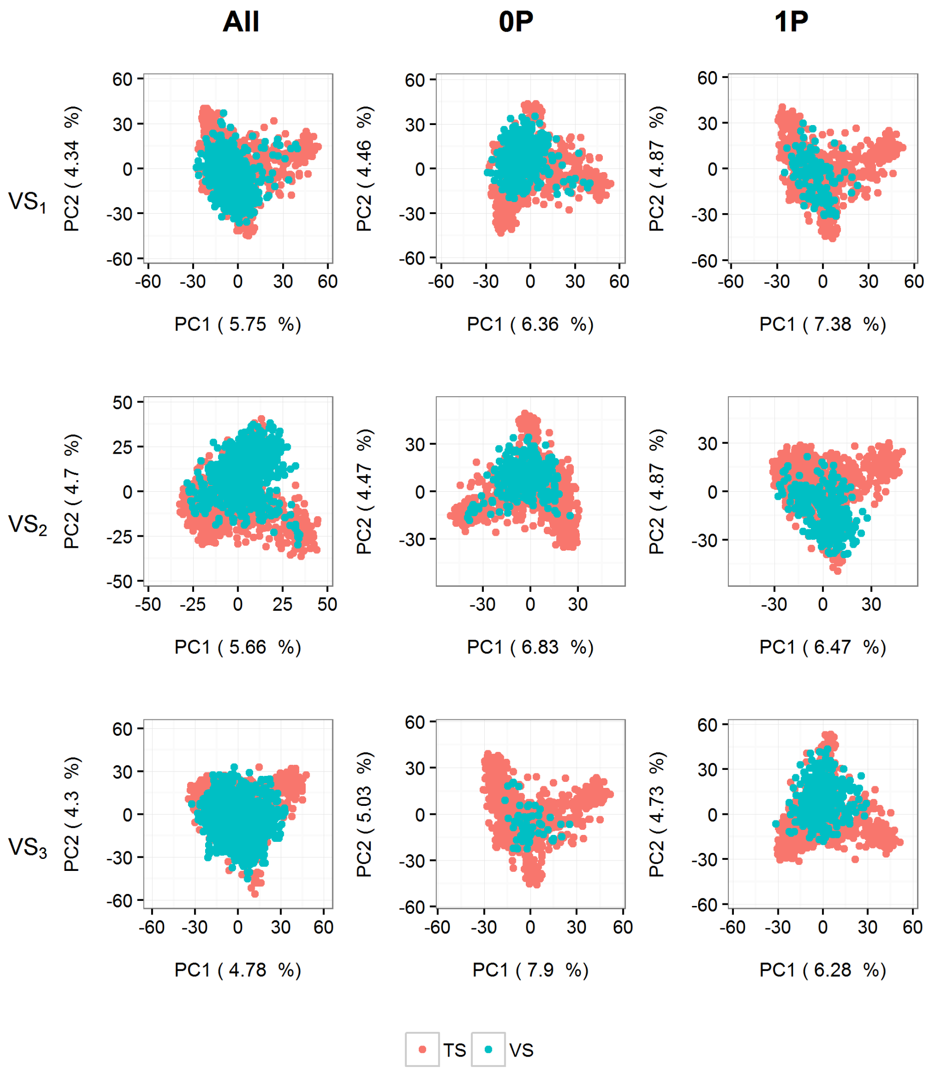

Figure S14: **Principal component (PC) plots for the German and Polish dataset between TS_1 and all VS.** TS_1 and relatedness scenarios (All-, 0P- and 1P-scenarios) for VS_1, VS_2 and VS_3. TS_1:GCA1-2009 + GCA2-2010 + GCA3-2011, VS_1:GCA1-2012, VS_2:GCA1-2013, VS_3:GCA1-2014.

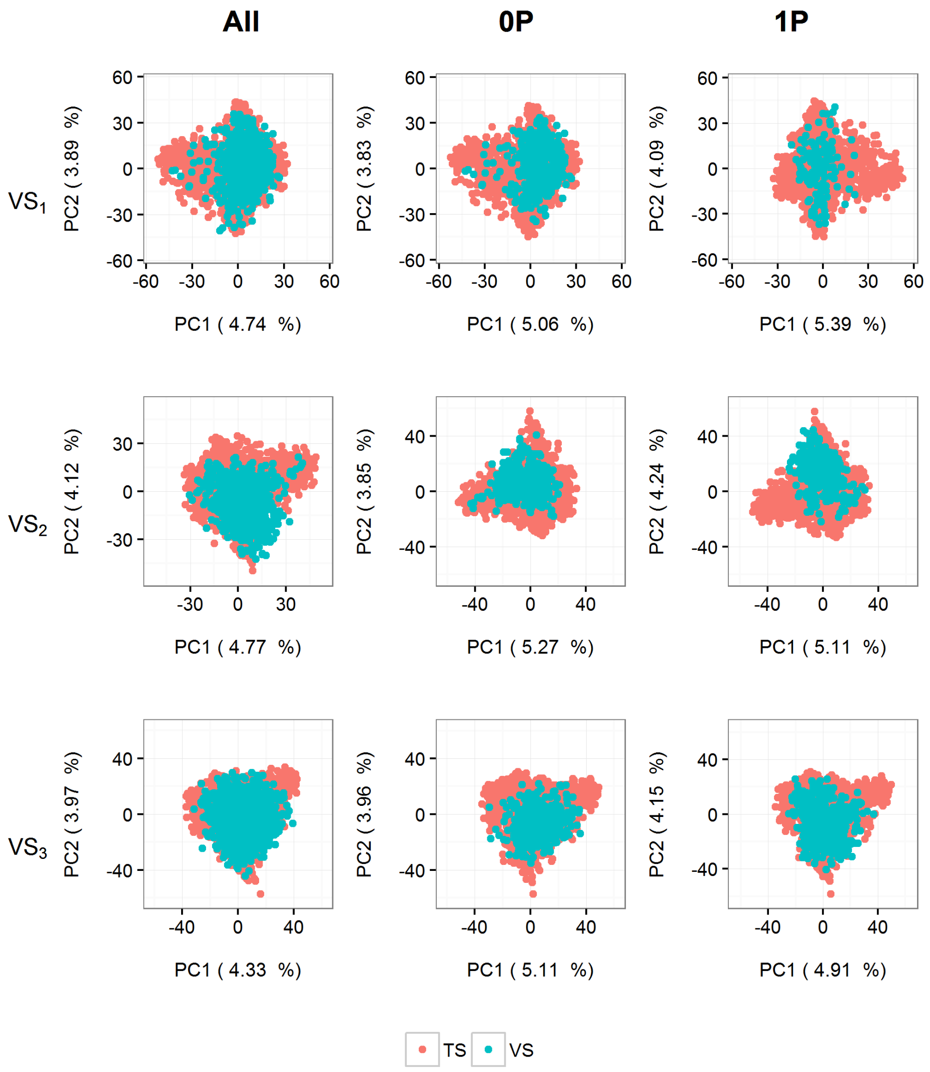

Figure S15: **Principal component (PC) plots for the German and Polish dataset between TS_2 and all VS.** TS_2 and relatedness scenarios (All-, 0P- and 1P-scenarios) for VS_1, VS_2 and VS_3. TS_2:GCA1-2009 + GCA2-2010 + GCA1-2010 + GCA2-2011, VS_1:GCA1-2012, VS_2:GCA1-2013, VS_3:GCA1-2014.

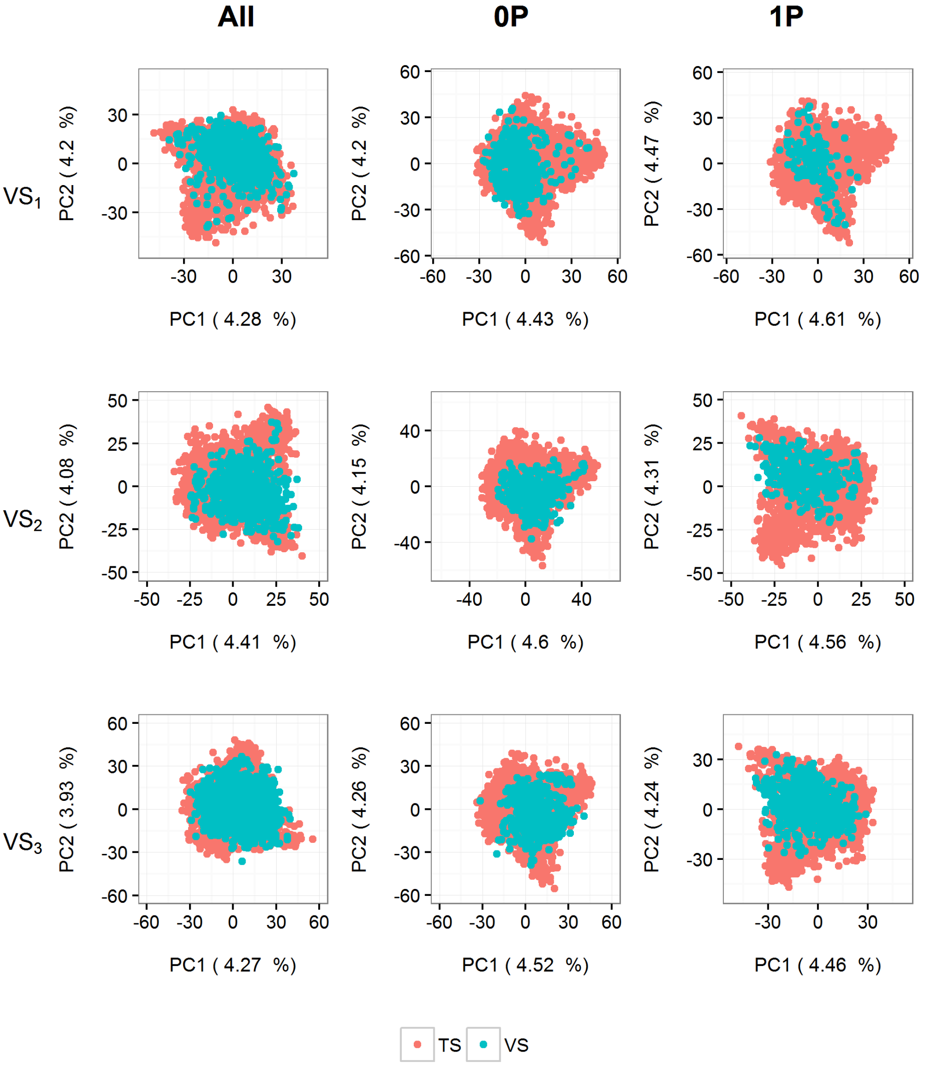

Figure S16: **Principal component (PC) plots for the German and Polish dataset between TS_3 and all VS.** TS_3 and relatedness scenarios (All-, 0P- and 1P-scenarios) for VS_1, VS_2 and VS_3. TS_3:GCA1-2009 + GCA2-2010 + GCA3-2011 + GCA1-2010 + GCA2-2011 + GCA3-2012 + GCA1-2011 + GCA2-2012 + GCA3-2013, VS_1:GCA1-2012, VS_2:GCA1-2013, VS_3:GCA1-2014.

7 Euclidean distance

Table S11: Means of Euclidean distance between all TS and VS combinations of the three datasets: German (GER), Polish (PL) and German and Polish (GER&PL), with All-, 0P- and 1P-scenarios.

		GER&PL			GER			PL		
		TS_1	TS_2	TS_3	TS_1	TS_2	TS_3	TS_1	TS_2	TS_3
VS_1	All	101.089	101.596	101.847	99.173	99.524	100.114	101.977	102.388	102.533
	0P	100.923	101.551	101.854	98.226	99.073	99.862	102.339	102.616	102.749
	1P	100.080	101.175	101.590	97.247	98.536	99.460	101.774	102.347	102.609
VS_2	All	100.839	101.453	101.764	98.168	98.826	99.624	102.170	102.726	102.883
	0P	100.265	101.187	101.613	96.860	98.365	99.368	102.238	102.654	102.840
	1P	100.657	101.428	101.771	97.543	98.509	99.452	102.321	102.781	102.928
VS_3	All	100.162	100.868	101.222	98.271	98.815	99.511	100.783	101.792	102.039
	0P	99.758	100.944	101.387	95.601	98.076	99.126	101.879	102.358	102.562
	1P	100.417	101.131	101.511	97.548	98.800	99.593	101.687	102.349	102.544
